# Simplified entanglement swapping protocol for the quantum Internet

**DOI:** 10.1038/s41598-023-49326-4

**Published:** 2023-12-11

**Authors:** Mario Mastriani

**Affiliations:** 1 Knight Foundation School of Computing and Information Sciences, Florida International University, 11200 S.W. 8th Street, Miami, FL 33199 USA; 2 Qubit Reset LLC, 2875 NE 191 suite 801, Aventura, FL 33180 USA

**Keywords:** Physics, Optics and photonics, Optical physics

## Abstract

In this study, a simplified version of the entanglement-swapping protocol, commonly used in the deployment of quantum networks, is presented. Quantum repeaters are essential in extending the range of quantum networks, especially when they are implemented through the laying of optical fiber. The simplified version of the entanglement-swapping protocol does not require the use of unitary transforms to finish characterizing the shared Bell state at both ends to be connected, as happens in the traditional version of the protocol, facilitating and reducing costs in quantum repeater implementations. Both a theoretical demonstration and an experimental one on an optical table, based on two revealing experiments, show the excellent performance of the presented protocol.

## Introduction

Entanglement^1–3^ constitutes one of the two scenarios where special relativity^4^ and quantum mechanics^5^ confront each other in a fierce fight, these being two of the main pillars of physics. This is the reason why, even today, the intrinsic springs of what, without a doubt, constitutes the most interesting and useful phenomenon in physics, i.e., entanglement, are unknown in all its magnitude. However, even with incomplete knowledge of it, the twenty-first century has become the moment of the birth of the so-called quantum information science^6^, of which quantum computation^7–11^, and quantum communications^12–15^ represent the most promising verticals, both having entanglement as an essential tool for their development. The advances produced in this area intrinsically imply small and important steps, due to the need to avoid a certain number of obstacles grouped in a family of no-go theorems^6^, of which the most conspicuous is without a doubt the no-cloning theorem^16^.

Of all the quantum communications protocols developed to date, quantum teleportation and super-dense coding^17^ are the first and most important. Countless efforts have been made during the last two decades to extend the applicability of these protocols to:

- transmit multiple qubits at the same time^18,19^,
- transmit the qubits in both directions^20–26^, and
- simplify its physical implementation^27–31^.

All these efforts aim to improve the performance of transmission protocols for their application in the future network of networks, i.e., the quantum Internet^32–41^.

In the case of this last option, a recurring concern, that arises in people who are not dedicated to technological aspects, is summarized in the following question: if the quantum internet will be implemented exclusively optically, then, what is the main difference with the optical implementations of the current Internet that justify such an investment in a new infrastructure? The answer is very simple, the security that the quantum version of the internet will offer using the laws of quantum mechanics^5^ to preserve the integrity and privacy of the information. This attribute is what will make the difference between both versions of the Internet, given that quantum cryptography protocols^42,43^ are the only ones that will be prepared to resist future attacks by quantum computers^6–11^, which use, for example, the famous Shor’s algorithm^44^, which allows factoring an integer, the basis of the cryptographic algorithm of Rivest, Shamir, and Adleman (RSA)^45^, in polynomial time.

Among the families of quantum cryptography algorithms with the best performance, we can highlight the following technologies:

- quantum key distribution (QKD)^46–49^, with and without entangled photons, where both ends of the quantum channel share a key to encrypt and decrypt the Information that is transmitted by the classical channel,
- quantum secure direct communications (QSDC)^50–64^, with and without entangled photons, which does not use a key to encrypt/decrypt the shared information, with all that this means from the point of view of security in quantum cryptography^42,43^, positioning itself as the family of protocols with the most future in quantum communications^12–15^, in general, and the future quantum internet^32–41^, in particular,
- quantum secret sharing (QSS)^65^, which uses entangled photon configurations of the Greenberger–Horne–Zeilinger (GHZ) type^1–3,6–8^, and does not use keys to encode the information; and
- quantum key secure communication (QKSC)^19^, which also uses entangled photon configurations of the GHZ type, also does not use keys and is fundamentally based on a version of the super-dense coding protocol extended to *N* qubits, where *N* is an arbitrary number.

Depending on the quantum cryptography protocol and whether it works with continuous variables, the pre-existing infrastructure of the current internet can be reused in its new quantum version.

Another aspect as important as security is the range of the future quantum internet, which must be extended using quantum repeaters, both in fiber optic runs^66^ and in free-space implementations employing CubeSats in the so-called low-earth-orbits (LEO)^67,68^.

Quantum repeaters are based on the entanglement-swapping protocol^69–75^, which has a series of drawbacks for its efficient implementation. These drawbacks are fundamentally represented using multiple classical channels, which distribute classical disambiguation bits that activate or deactivate unitary transforms at both ends to be connected by the quantum repeater. Synchronization problems in the activation of both unitary transforms, located at both ends to be connected, must be compensated with intensive use of quantum memories^76^. This becomes even more complicated as more and more quantum repeaters are incorporated to appropriately extend the link range. Therefore, any simplification in the implementation of the entanglement-swapping protocol, such as the one presented in the present study, will be very welcome.

This paper comprises various sections as follows. The next section explains the theoretical foundations of the simplified entanglement swapping protocol and its most notable differences from the original protocol. The “Bell state change test” section is dedicated to explaining the theory of the first experiment that will verify the correct functioning of the new protocol, while in the “Bell test from two independent sources of entangled photons” section, we do the same with the second prepared experiment. In the section “Optical table”, we will implement both tests on the optical table. In the “Discussions about the outcomes” section, we discuss the results of the proposed protocols by showing the fidelity of the circuits. Finally, we end our paper with a brief conclusion in the last section.

## Simplified entanglement swapping

**Figure 1:**
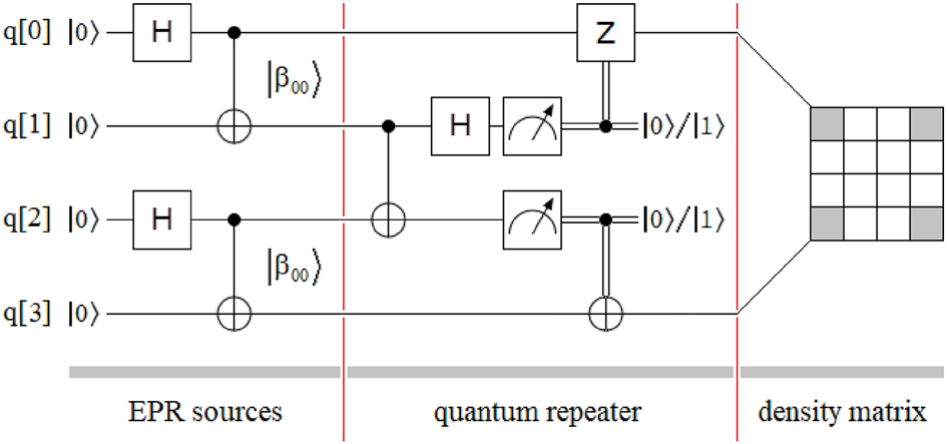
Sketch of the entanglement swapping protocol composed of three well-defined sectors: two independent EPR sources of the $\left| {\beta_{00} } \right\rangle$-type, one quantum repeater between both sources, and the density matrix of the final state $\rho_{{\left| {\beta_{00} } \right\rangle }}$, where a gray element represents a value of 0.5, while a white element is equal to a value of 0.

Entanglement swapping protocol^69–75^ is represented in Fig. 1, which shows two independent EPR (Einstein–Podolsky–Rosen)^6^ sources of entanglement photons, one quantum repeater^66^, and the resulting density matrix between the extreme qubits q[0] and q[3]. The first EPR source is allocated between the qubits q[0] and q[1], while the second one is between the qubits q[2] and q[3]. At the output of each crystal used to generate the entangled photons, the Bell^6^ state obtained is of the following type:

1 $$\left| {\Phi^{ + } } \right\rangle = {1 \mathord{\left/ {\vphantom {1 {\sqrt 2 }}} \right. \kern-0pt} {\sqrt 2 }}\left( {\left| {HH} \right\rangle + e^{i\phi } \left| {VV} \right\rangle } \right),$$

where $$\left| H \right\rangle = \left| 0 \right\rangle = \left[ {\begin{array}{*{20}c} 1 \\ 0 \\ \end{array} } \right]$$, $$\left| V \right\rangle = \left| 1 \right\rangle = \left[ {\begin{array}{*{20}c} 0 \\ 1 \\ \end{array} } \right]$$, and the relative phase ϕ depends mainly on the phase-matching conditions and the width of the crystal used^77^. However, in order not to complicate the subsequent theoretical deduction of the new protocol, the Bell state considered here will be the following:

2 $$\left| {\beta_{00} } \right\rangle = {1 \mathord{\left/ {\vphantom {1 {\sqrt 2 }}} \right. \kern-0pt} {\sqrt 2 }}\left( {\left| {00} \right\rangle + \left| {11} \right\rangle } \right) = CNOT\left( {H \otimes I} \right)\left| {00} \right\rangle = \left[ {\begin{array}{*{20}c} {{1 \mathord{\left/ {\vphantom {1 {\sqrt 2 }}} \right. \kern-0pt} {\sqrt 2 }}} & 0 & 0 & {{1 \mathord{\left/ {\vphantom {1 {\sqrt 2 }}} \right. \kern-0pt} {\sqrt 2 }}} \\ \end{array} } \right]^{T} ,$$

where $$CNOT = \left[ {\begin{array}{*{20}c} 1 & 0 & 0 & 0 \\ 0 & 1 & 0 & 0 \\ 0 & 0 & 0 & 1 \\ 0 & 0 & 1 & 0 \\ \end{array} } \right]$$ is the Feynman’s gate^6^, $$H = \frac{1}{\sqrt 2 }\left[ {\begin{array}{*{20}c} 1 & {\;\;1} \\ 1 & { - 1} \\ \end{array} } \right]$$ is the Hadamard’s gate^6^, $$I = \left[ {\begin{array}{*{20}c} 1 & 0 \\ 0 & 1 \\ \end{array} } \right]$$ is the identity matrix, (·)^*T*^ means transpose of (·), and ⨂ is the Kronecker’s product^6^.

As we will see in the experiments on the optical table, all measurement modules will be implemented using polarizing beam-splitting (PBS)^77^, and avalanche photodiodes (APD), and configured according to the H/V basis. Since we will use PBSs of the best quality on the market, and we consider that we will manipulate Bell states of the type of Eq. (2), we expect quasi-perfectly equiprobable outputs for both faces of the PBSs used in configurations like that of Fig. 1:

3 $$M = \left\{ {\begin{array}{*{20}c} {P_{h} } \\ {50\% } \\ \end{array} \begin{array}{*{20}c} , \\ {} \\ \end{array} \begin{array}{*{20}c} {P_{v} } \\ {50\% } \\ \end{array} \;} \right\} = \left\{ {\begin{array}{*{20}c} {\left| H \right\rangle \left\langle H \right|} \\ {50\% } \\ \end{array} \begin{array}{*{20}c} , \\ {} \\ \end{array} \begin{array}{*{20}c} {\left| V \right\rangle \left\langle V \right|} \\ {50\% } \\ \end{array} } \right\} = \left\{ {\begin{array}{*{20}c} {\left[ {\begin{array}{*{20}c} 1 & 0 \\ 0 & 0 \\ \end{array} } \right]} \\ {50\% } \\ \end{array} ,\begin{array}{*{20}c} {\left[ {\begin{array}{*{20}c} 0 & 0 \\ 0 & 1 \\ \end{array} } \right]} \\ {50\% } \\ \end{array} } \right\}$$

where *M* is the measurement operator, *P*_*h*_ is the horizontal polarization operator, and *P*_*v*_ is the vertical polarization operator.

In Fig. 1, the intervention of the quantum repeater gives rise to transitivity (q[0] *is entangled with* q[1], and q[2] is entangled with q[3] ⟹ q[0] is entangled with q[3], but q[1] is not entangled with q[2]), where the original entangled states at the output of each EPR source end up linking in a new unique and final Bell state, between the qubits furthest from the configuration, which is concomitant with the idea of extending the range of the entanglement so that the quantum network reaches where it is necessary.

From now on, in all theoretical figures, single lines represent quantum wires, while double lines will be classical wires.

The resulting density matrix, between the extreme qubits q[0] and q[3], will be identical to the density matrices of the original EPR sources between the respective qubits q[0] and q[1], and q[2] and q[3]. In consequence, considering Eq. (2), yields:

4 $$\rho_{{\left| {\beta_{00} } \right\rangle }} = \left| {\beta_{00} } \right\rangle \left\langle {\beta_{00} } \right| = \left[ {\begin{array}{*{20}c} {{1 \mathord{\left/ {\vphantom {1 {\sqrt 2 }}} \right. \kern-0pt} {\sqrt 2 }}} \\ 0 \\ 0 \\ {{1 \mathord{\left/ {\vphantom {1 {\sqrt 2 }}} \right. \kern-0pt} {\sqrt 2 }}} \\ \end{array} } \right]\left[ {\begin{array}{*{20}c} {{1 \mathord{\left/ {\vphantom {1 {\sqrt 2 }}} \right. \kern-0pt} {\sqrt 2 }}} & {\quad 0} & {\quad 0} & {{{\quad 1} \mathord{\left/ {\vphantom {{\quad 1} {\sqrt 2 }}} \right. \kern-0pt} {\sqrt 2 }}} \\ \end{array} } \right] = \left[ {\begin{array}{*{20}c} {{1 \mathord{\left/ {\vphantom {1 2}} \right. \kern-0pt} 2}} & {\quad 0} & {\quad 0} & {{{\quad 1} \mathord{\left/ {\vphantom {{\quad 1} 2}} \right. \kern-0pt} 2}} \\ 0 & {\quad 0} & {\quad 0} & {\quad 0} \\ 0 & {\quad 0} & {\quad 0} & {\quad 0} \\ {{1 \mathord{\left/ {\vphantom {1 2}} \right. \kern-0pt} 2}} & {\quad 0} & {\quad 0} & {{{\quad 1} \mathord{\left/ {\vphantom {{\quad 1} 2}} \right. \kern-0pt} 2}} \\ \end{array} } \right].$$

Therefore, in the following theoretical figures, a gray element of the density matrix represents a value of 0.5, while a white element is equal to a value of 0.

**Figure 2:**
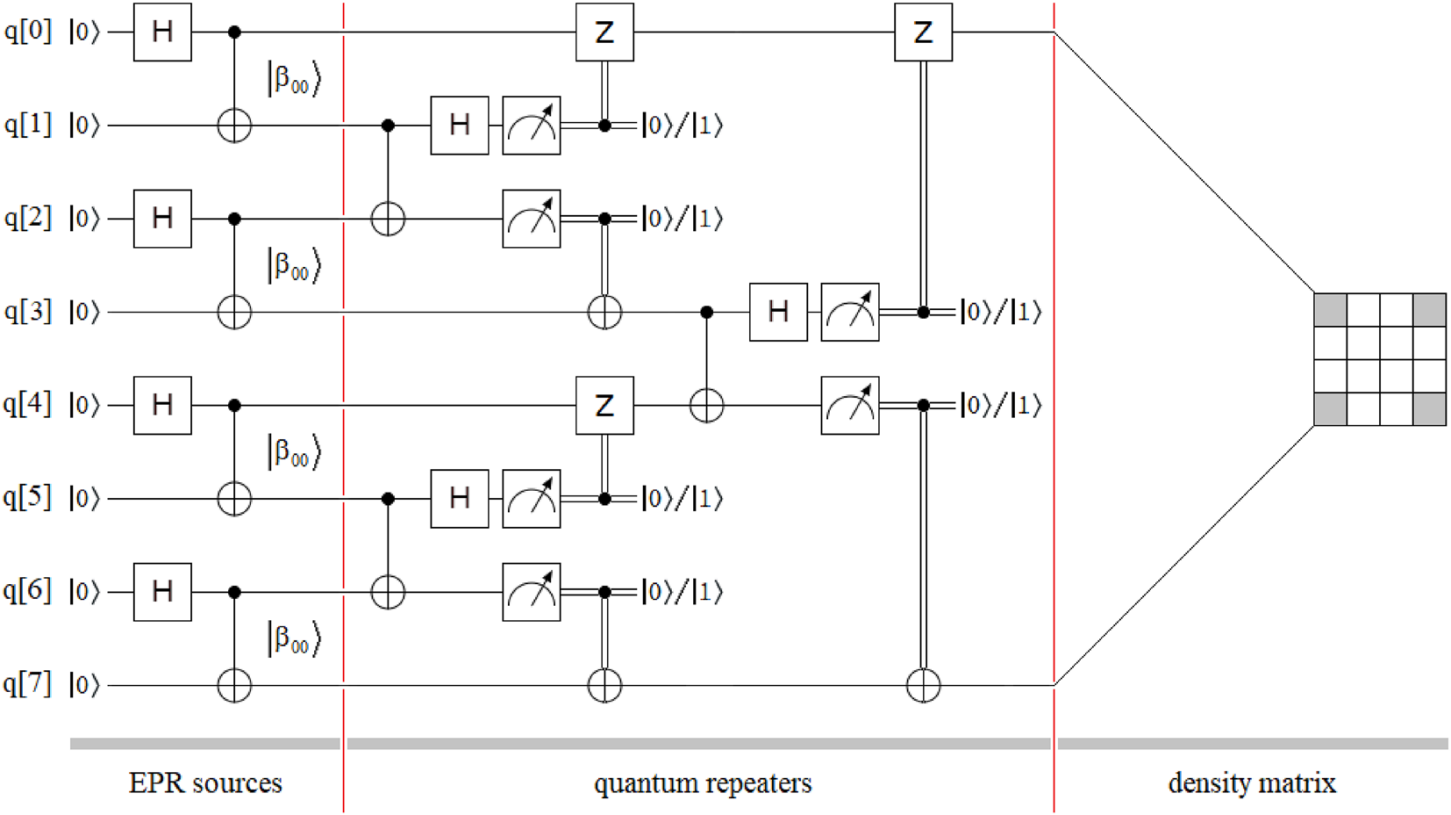
Sketch of the entanglement swapping protocol composed of three well-defined sectors: four independent EPR sources of the $\left| {\beta_{00} } \right\rangle$-type, three quantum repeaters between those sources, and the density matrix of the final state $\rho_{{\left| {\beta_{00} } \right\rangle }}$. The application of the protocol is carried out by grouping the states of the $\left| {\beta_{00} } \right\rangle$-type in pairs until reaching a single final state between the extreme qubits q[0] and q[7], thus reaching the maximum extension of the entanglement range for this configuration.

Figure 2 shows a more complete intervention of the entanglement swapping protocol when it is used with four independent EPR sources, then intervening three quantum repeaters, but with identical results to the previous case, given that we obtain a density matrix like that of Eq. (4) between the most extreme qubits, i.e., q[0] and q[7]. In this case, we are in the presence of a double transitivity, because the application of the protocol takes place involving pairs of EPR sources. Thus, q[0] is entangled with q[1], and q[2] is entangled with q[3] ⟹ q[0] is entangled with q[3]; but q[1] is not entangled with q[2], and q[4] is entangled with q[5], and q[6] is entangled with q[7] ⟹ q[4] is entangled with q[7]; but q[5] is not entangled with q[6], ⟹ q[0] is entangled with q[7]; but q[3] is not entangled with q[4].

**Figure 3:**
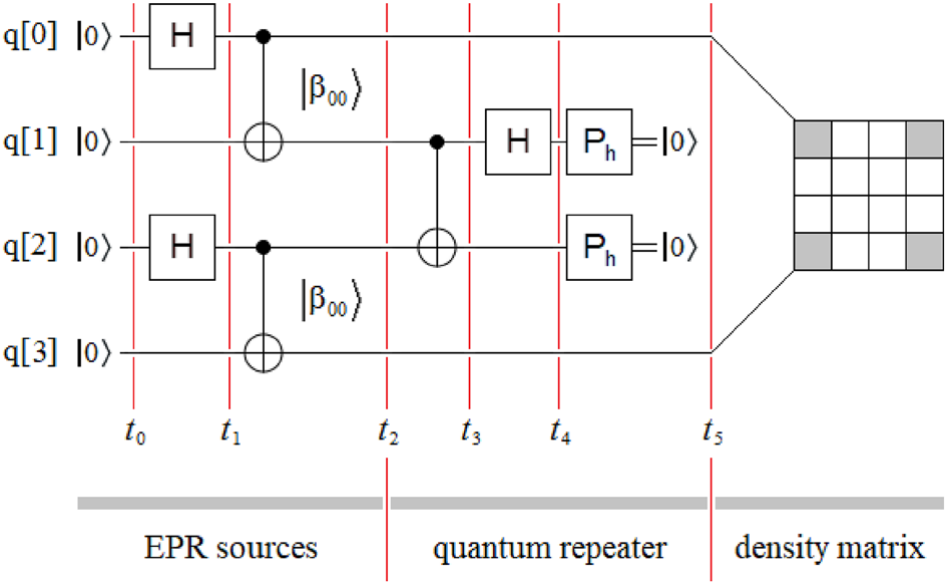
Sketch of the proposed entanglement swapping protocol also composed of three well-defined sectors: two independent EPR sources of the $\left| {\beta_{00} } \right\rangle$-type, one quantum repeater between both sources and the density matrix of the final state $\rho_{{\left| {\beta_{00} } \right\rangle }}$, where the only difference with the scheme in Fig. 1 lies in the quantum repeater.

Figure 3 shows the simplified entanglement swapping protocol applied to the simplest possible case, which is equivalent to that of Fig. 1 for the original version of the protocol. In this case, we will proceed to the theoretical deduction of the protocol to verify its performance. For this, we calculate the joint state of the four qubits at each of the six instants $\left( {t_{i} \forall i \in \left[ {0,5} \right]} \right)$ indicated in Fig. 1:

5a $$\left| {\psi \left( {t_{0} } \right)} \right\rangle = \left| {0000} \right\rangle ,$$

5b $$\begin{gathered} \left| {\psi \left( {t_{1} } \right)} \right\rangle = \left( {H \otimes I \otimes H \otimes I} \right)\left| {\psi \left( {t_{0} } \right)} \right\rangle \hfill \\ \quad \quad \quad = \left( {\frac{1}{\sqrt 2 }\left[ {\begin{array}{*{20}c} 1 & {\quad 1} \\ 1 & {\quad - 1} \\ \end{array} } \right] \otimes \left[ {\begin{array}{*{20}c} 1 & 0 \\ 0 & 1 \\ \end{array} } \right] \otimes \frac{1}{\sqrt 2 }\left[ {\begin{array}{*{20}c} 1 & {\quad 1} \\ 1 & {\quad - 1} \\ \end{array} } \right] \otimes \left[ {\begin{array}{*{20}c} 1 & {\quad 0} \\ 0 & {\quad 1} \\ \end{array} } \right]} \right)\left| {0000} \right\rangle \hfill \\ \quad \quad \quad = \left[ {\begin{array}{*{20}c} {\begin{array}{*{20}c} {\begin{array}{*{20}c} {\begin{array}{*{20}c} {\begin{array}{*{20}c} {{1 \mathord{\left/ {\vphantom {1 2}} \right. \kern-0pt} 2}} & 0 & {{1 \mathord{\left/ {\vphantom {1 2}} \right. \kern-0pt} 2}} & 0 \\ \end{array} } & 0 & 0 & 0 \\ \end{array} } & 0 & {{1 \mathord{\left/ {\vphantom {1 2}} \right. \kern-0pt} 2}} & 0 \\ \end{array} } & {{1 \mathord{\left/ {\vphantom {1 2}} \right. \kern-0pt} 2}} & 0 & 0 \\ \end{array} } & 0 & 0 & 0 \\ \end{array} } \right]^{T} \hfill \\ \quad \quad \quad = \left| + \right\rangle \otimes \left| 0 \right\rangle \otimes \left| + \right\rangle \otimes \left| 0 \right\rangle , \hfill \\ \end{gathered}$$

where $$\left| + \right\rangle = \left[ {\begin{array}{*{20}c} {{1 \mathord{\left/ {\vphantom {1 {\sqrt 2 }}} \right. \kern-0pt} {\sqrt 2 }}} & {{1 \mathord{\left/ {\vphantom {1 {\sqrt 2 }}} \right. \kern-0pt} {\sqrt 2 }}} \\ \end{array} } \right]^{T}$$,

5c $$\begin{gathered} \left| {\psi \left( {t_{2} } \right)} \right\rangle = \left( {CNOT \otimes CNOT} \right)\left| {\psi \left( {t_{1} } \right)} \right\rangle \hfill \\ \quad \quad \quad = \left( {\left[ {\begin{array}{*{20}c} 1 & {\quad 0} & {\quad 0} & {\quad 0} \\ 0 & {\quad 1} & {\quad 0} & {\quad 0} \\ 0 & {\quad 0} & {\quad 0} & {\quad 1} \\ 0 & {\quad 0} & {\quad 1} & {\quad 0} \\ \end{array} } \right] \otimes \left[ {\begin{array}{*{20}c} 1 & {\quad 0} & {\quad 0} & {\quad 0} \\ 0 & {\quad 1} & {\quad 0} & {\quad 0} \\ 0 & {\quad 0} & {\quad 0} & {\quad 1} \\ 0 & {\quad 0} & {\quad 1} & {\quad 0} \\ \end{array} } \right]} \right)\left| { + 0 + 0} \right\rangle \hfill \\ \quad \quad \quad = \left[ {\begin{array}{*{20}c} {\begin{array}{*{20}c} {\begin{array}{*{20}c} {\begin{array}{*{20}c} {\begin{array}{*{20}c} {{1 \mathord{\left/ {\vphantom {1 2}} \right. \kern-0pt} 2}} & 0 & 0 & {{1 \mathord{\left/ {\vphantom {1 2}} \right. \kern-0pt} 2}} \\ \end{array} } & 0 & 0 & 0 \\ \end{array} } & 0 & 0 & 0 \\ \end{array} } & 0 & 0 & {{1 \mathord{\left/ {\vphantom {1 2}} \right. \kern-0pt} 2}} \\ \end{array} } & 0 & 0 & {{1 \mathord{\left/ {\vphantom {1 2}} \right. \kern-0pt} 2}} \\ \end{array} } \right]^{T} \hfill \\ \quad \quad \quad = \left| {\beta_{00} } \right\rangle \otimes \left| {\beta_{00} } \right\rangle , \hfill \\ \end{gathered}$$

5d $$\begin{gathered} \left| {\psi \left( {t_{3} } \right)} \right\rangle = \left( {I \otimes CNOT \otimes I} \right)\left| {\psi \left( {t_{2} } \right)} \right\rangle \hfill \\ \quad \quad \quad = \left( {\left[ {\begin{array}{*{20}c} 1 & {\quad 0} \\ 0 & {\quad 1} \\ \end{array} } \right] \otimes \left[ {\begin{array}{*{20}c} 1 & {\quad 0} & {\quad 0} & {\quad 0} \\ 0 & {\quad 1} & {\quad 0} & {\quad 0} \\ 0 & {\quad 0} & {\quad 0} & {\quad 1} \\ 0 & {\quad 0} & {\quad 1} & {\quad 0} \\ \end{array} } \right] \otimes \left[ {\begin{array}{*{20}c} 1 & {\quad 0} \\ 0 & {\quad 1} \\ \end{array} } \right]} \right)\left| {\beta_{00} \beta_{00} } \right\rangle \hfill \\ \quad \quad \quad = \left[ {\begin{array}{*{20}c} {\begin{array}{*{20}c} {\begin{array}{*{20}c} {\begin{array}{*{20}c} {\begin{array}{*{20}c} {{1 \mathord{\left/ {\vphantom {1 2}} \right. \kern-0pt} 2}} & 0 & 0 & {{1 \mathord{\left/ {\vphantom {1 2}} \right. \kern-0pt} 2}} \\ \end{array} } & 0 & 0 & 0 \\ \end{array} } & 0 & 0 & 0 \\ \end{array} } & 0 & 0 & 0 \\ \end{array} } & {{1 \mathord{\left/ {\vphantom {1 2}} \right. \kern-0pt} 2}} & {{1 \mathord{\left/ {\vphantom {1 2}} \right. \kern-0pt} 2}} & 0 \\ \end{array} } \right]^{T} , \hfill \\ \end{gathered}$$

5e $$\begin{gathered} \left| {\psi \left( {t_{4} } \right)} \right\rangle = \left( {I \otimes H \otimes I \otimes I} \right)\left| {\psi \left( {t_{3} } \right)} \right\rangle \hfill \\ \quad \quad \quad = \left( {\left[ {\begin{array}{*{20}c} 1 & {\quad 0} \\ 0 & {\quad 1} \\ \end{array} } \right] \otimes \frac{1}{\sqrt 2 }\left[ {\begin{array}{*{20}c} 1 & {\quad 1} \\ 1 & {\quad - 1} \\ \end{array} } \right] \otimes \left[ {\begin{array}{*{20}c} 1 & {\quad 0} & {\quad 0} & {\quad 0} \\ 0 & {\quad 1} & {\quad 0} & {\quad 0} \\ 0 & {\quad 0} & {\quad 1} & {\quad 0} \\ 0 & {\quad 0} & {\quad 0} & {\quad 1} \\ \end{array} } \right]} \right)\left| {\psi \left( {t_{3} } \right)} \right\rangle \hfill \\ \quad \quad \quad = \left[ {\begin{array}{*{20}c} {\begin{array}{*{20}c} {\begin{array}{*{20}c} {\begin{array}{*{20}c} {\begin{array}{*{20}c} 1 & 0 & 0 & 1 \\ \end{array} } & 1 & 0 & 0 \\ \end{array} } & 1 & 0 & 1 \\ \end{array} } & 1 & 0 & 0 \\ \end{array} } & { - 1} & { - 1} & 0 \\ \end{array} } \right]^{T} {{\sqrt 2 } \mathord{\left/ {\vphantom {{\sqrt 2 } 4}} \right. \kern-0pt} 4}, \hfill \\ \end{gathered}$$

and

5f $$\begin{gathered} \left| {\psi \left( {t_{5} } \right)} \right\rangle = \left( {I \otimes P_{h} \otimes P_{h} \otimes I} \right)\left| {\psi \left( {t_{4} } \right)} \right\rangle \hfill \\ \quad \quad \quad = \left( {\left[ {\begin{array}{*{20}c} 1 & 0 \\ 0 & 1 \\ \end{array} } \right] \otimes \left[ {\begin{array}{*{20}c} {\quad 1} & {\quad 0} \\ {\quad 0} & {\quad 0} \\ \end{array} } \right] \otimes \left[ {\begin{array}{*{20}c} 1 & {\quad 0} \\ 0 & {\quad 0} \\ \end{array} } \right] \otimes \left[ {\begin{array}{*{20}c} 1 & {\quad 0} \\ 0 & {\quad 1} \\ \end{array} } \right]} \right)\left| {\psi \left( {t_{4} } \right)} \right\rangle \hfill \\ \quad \quad \quad = \left[ {\begin{array}{*{20}c} {\begin{array}{*{20}c} {\begin{array}{*{20}c} {\begin{array}{*{20}c} {\begin{array}{*{20}c} 1 & {\quad 0} & {\quad 0} & {\quad 0} \\ \end{array} } & {\quad 0} & {\quad 0} & {\quad 0} \\ \end{array} } & {\quad 0} & {\quad 0} & {\quad 1} \\ \end{array} } & {\quad 0} & {\quad 0} & {\quad 0} \\ \end{array} } & {\quad 0} & {\quad 0} & {\quad 0} \\ \end{array} } \right]^{T} {{\sqrt 2 } \mathord{\left/ {\vphantom {{\sqrt 2 } 4}} \right. \kern-0pt} 4}. \hfill \\ \end{gathered}$$

The strange final state resulting from Eq. (5f) is the same one obtained in the far right of Fig. 3, where we have a shared state of the $\left| {\beta_{00} } \right\rangle$-type between the qubits q[0] and q[3], while the central qubits remain at $\left| 0 \right\rangle$. We also noticed a drop of 3dB, i.e., ½, in the final state modulus due to the action of both horizontal polarizers *P*_*h*_. However, this decay only takes place in the central qubits and not in the extreme ones, i.e., those that share the $\left| {\beta_{00} } \right\rangle$ state, which is complete and without attenuation.

In other words, at time *t*_5_ in Fig. 3 we obtain the same identity matrix as that of the original protocol in Figs. 1 and 2, with gray elements at ½ and white elements at 0.

Comparing both versions of the entanglement swapping protocol, see Figs. 1 and 3, we can observe the simplicity of the new version compared to the traditional one.

In Appendix A, we discuss the impact of non-maximal entangled states because of the presence of noise in the quantum channel, which degrades the fidelity of the entanglement.

**Figure 4:**
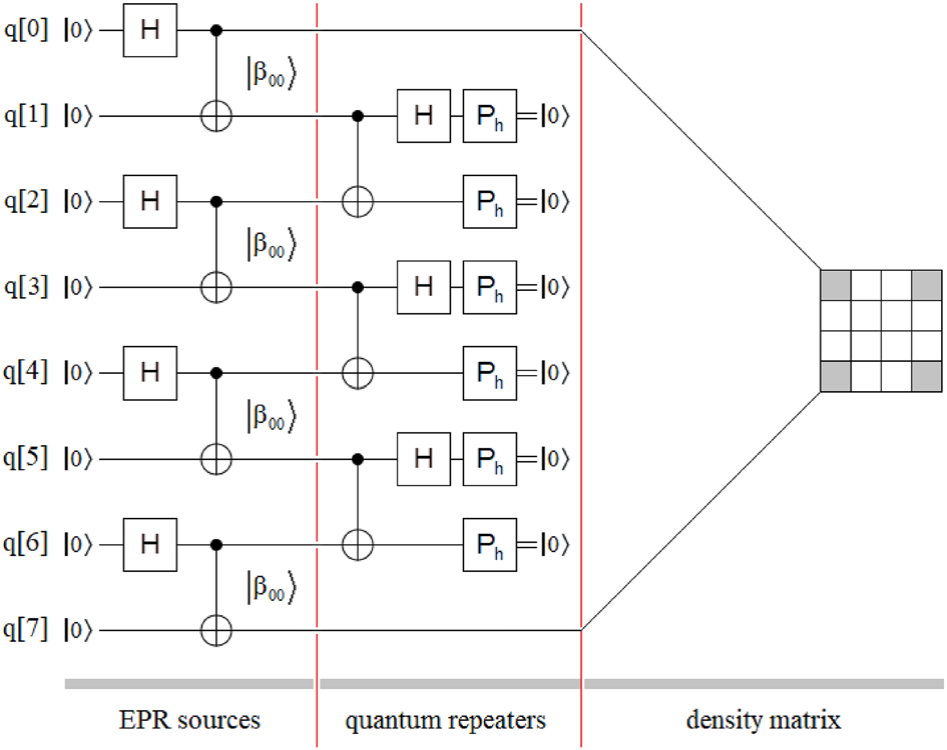
Sketch of the new entanglement swapping protocol is also composed of three well-defined sectors: four independent EPR sources of the $\left| {\beta_{00} } \right\rangle$-type, three quantum repeaters between those sources, and the density matrix of the final state $\rho_{{\left| {\beta_{00} } \right\rangle }}$. As in Fig. 2, the application of the new protocol is carried out by grouping the states of the $\left| {\beta_{00} } \right\rangle$-type in pairs until reaching a single final state between the extreme qubits q[0] and q[7], thus reaching the maximum extension of the entanglement range for this configuration.

Figure 4 shows an extension of the new protocol of Fig. 3, which inherits the simplicity in the transitivity from the last one, but for the case of four independent EPR sources and three quantum repeaters. As in Figs. 1 and 2, in Figs. 3 and 4 we can see three well-defined areas: EPR sources, quantum repeaters, and density matrix (outcomes). In Fig. 4, the total absence of classic channels, as well as the unitary transforms associated with them, simplifies implementation, lowers costs, and reduces the time involved in the project. Finally, as we will see in the experiments on the optical table, the great regularity in the structure of the quantum repeater modules will be reflected in their optical implementation, where said implementation will be exclusively optical with all the advantages that this will entail for the future industry associated with the future quantum internet^32–41^.

## Bell state change test

Below is the theoretical description of the first experiment that will help us evaluate the performance of the new entanglement swapping protocol.

**Figure 5:**
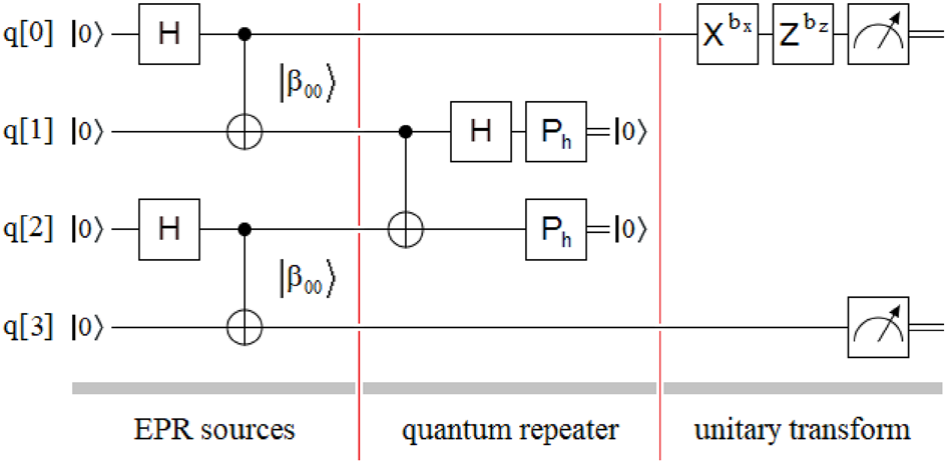
Bell state change test: the same configuration of Fig. 1 with a pair of Pauli gates {*X*, *Z*} with their respective activation bits {*b*_x_, *b*_z_} in the qubit q[0], after entanglement swapping.

In the upper right corner of Fig. 5, after carrying out the supposedly successful entanglement swapping by the new protocol, we introduce two gates, of the Pauli^6^ matrix type {*X*, *Z*}, in the qubit q[0]:

$$X = \left[ {\begin{array}{*{20}c} 0 & {\quad 1} \\ 1 & {\quad 0} \\ \end{array} } \right]$$, and

$$Z = \left[ {\begin{array}{*{20}c} 1 & {\quad 0} \\ 0 & {\quad - 1} \\ \end{array} } \right]$$,

Each with its respective activation bit, i.e., phase and parity bits {*b*_x_, *b*_z_}. Both gates will be implemented on the optical table as two half-wave plates (HWP).

The experiment consists of demonstrating that as a result of the action of the new protocol, two originally independent qubits, such as q[0] and q[3], are now maximally correlated, sharing a Bell state of the $\left| {\beta_{00} } \right\rangle$-type, in such a way that the activation of the *X* and *Z* gates will force a change of state in the Bell state between both qubits. This can be seen in detail in Table 1, where if we assume the success of the protocol, the qubits q[0] and q[3] will share a Bell state of the $\left| {\beta_{00} } \right\rangle$-type, for *b*_x_ = 0 and *b*_z_ = 0, i.e., both qubits are correlated. However, if, for example, we activate the parity bit *b*_x_, the gate X, we then go from the state of Bell $\left| {\beta_{00} } \right\rangle$ to the state of Bell $\left| {\beta_{01} } \right\rangle$, with the qubits q[0] and q[5] now being anti-correlated. The transitions between Bell states in Table 1 should be checked on the optical table by comparing and analyzing the correlations between the qubits q[0] and q[5].

**Table 1:** Maximally entangled states in terms of phase (*b*_*z*_) and parity (*b*_*x*_) bits.

*b*_*z*_	*b*_*x*_	$\left| {{\upbeta }_{{b_{z} b_{x} }} } \right\rangle$
0	0	$${{\left[ {\begin{array}{*{20}c} {1} & {0} & {0} & {1} \\ \end{array} } \right]^{{\text{T}}} } \mathord{\left/ {\vphantom {{\left[ {\begin{array}{*{20}c} {1} & {0} & {0} & {1} \\ \end{array} } \right]^{{\text{T}}} } {\sqrt {2} }}} \right. \kern-0pt} {\sqrt {2} }} = \left| {{\upbeta }_{{{00}}} } \right\rangle$$
1	0	$${{\left[ {\begin{array}{*{20}c} {1} & {0} & {0} & { - {1}} \\ \end{array} } \right]^{{\text{T}}} } \mathord{\left/ {\vphantom {{\left[ {\begin{array}{*{20}c} {1} & {0} & {0} & { - {1}} \\ \end{array} } \right]^{{\text{T}}} } {\sqrt {2} }}} \right. \kern-0pt} {\sqrt {2} }} = \left| {{\upbeta }_{{{10}}} } \right\rangle$$
0	1	$${{\left[ {\begin{array}{*{20}c} {0} & {1} & {1} & {0} \\ \end{array} } \right]^{{\text{T}}} } \mathord{\left/ {\vphantom {{\left[ {\begin{array}{*{20}c} {0} & {1} & {1} & {0} \\ \end{array} } \right]^{{\text{T}}} } {\sqrt {2} }}} \right. \kern-0pt} {\sqrt {2} }} = \left| {{\upbeta }_{{{01}}} } \right\rangle$$
1	1	$${{\left[ {\begin{array}{*{20}c} {0} & {1} & { - {1}} & {0} \\ \end{array} } \right]^{{\text{T}}} } \mathord{\left/ {\vphantom {{\left[ {\begin{array}{*{20}c} {0} & {1} & { - {1}} & {0} \\ \end{array} } \right]^{{\text{T}}} } {\sqrt {2} }}} \right. \kern-0pt} {\sqrt {2} }} = \left| {{\upbeta }_{{{11}}} } \right\rangle$$

As a theoretical check, if the final state of Eq. (5f) we first apply a SWAP gate between the states q[3] and q[2], and then another SWAP gate between the qubits q[2] and q[3], we will have:

6 $$\begin{gathered} \left| {\psi \left( {t_{6} } \right)} \right\rangle = \left( {I \otimes I \otimes SWAP} \right)\left( {I \otimes SWAP \otimes I} \right)\left| {\psi \left( {t_{5} } \right)} \right\rangle \hfill \\ \quad \quad \quad = \left( {\left[ {\begin{array}{*{20}c} 1 & 0 \\ 0 & 1 \\ \end{array} } \right] \otimes \left[ {\begin{array}{*{20}c} 1 & {\quad 0} \\ 0 & {\quad 1} \\ \end{array} } \right] \otimes \left[ {\begin{array}{*{20}c} 1 & {\quad 0} & {\quad 0} & {\quad 0} \\ 0 & {\quad 0} & {\quad 1} & {\quad 0} \\ 0 & {\quad 1} & {\quad 0} & {\quad 0} \\ 0 & {\quad 0} & {\quad 0} & {\quad 1} \\ \end{array} } \right]} \right)\left( {\left[ {\begin{array}{*{20}c} 1 & {\quad 0} \\ 0 & {\quad 1} \\ \end{array} } \right] \otimes \left[ {\begin{array}{*{20}c} 1 & {\quad 0} & {\quad 0} & {\quad 0} \\ 0 & {\quad 0} & {\quad 1} & {\quad 0} \\ 0 & {\quad 1} & {\quad 0} & {\quad 0} \\ 0 & {\quad 0} & {\quad 0} & {\quad 1} \\ \end{array} } \right] \otimes \left[ {\begin{array}{*{20}c} 1 & {\quad 0} \\ 0 & {\quad 1} \\ \end{array} } \right]} \right)\left| {\psi \left( {t_{5} } \right)} \right\rangle \hfill \\ \quad \quad \quad = \left[ {\begin{array}{*{20}c} {\begin{array}{*{20}c} {\begin{array}{*{20}c} {\begin{array}{*{20}c} {\begin{array}{*{20}c} 1 & 0 & 0 & 0 \\ \end{array} } & 0 & 0 & 0 \\ \end{array} } & 0 & 0 & 0 \\ \end{array} } & 0 & 0 & 1 \\ \end{array} } & 0 & 0 & 0 \\ \end{array} } \right]^{T} {{\sqrt 2 } \mathord{\left/ {\vphantom {{\sqrt 2 } 4}} \right. \kern-0pt} 4} \hfill \\ \quad \quad \quad = \left| {\beta_{00} } \right\rangle \left| {00} \right\rangle {{\sqrt 2 } \mathord{\left/ {\vphantom {{\sqrt 2 } 4}} \right. \kern-0pt} 4}. \hfill \\ \end{gathered}$$

If we now activate the parity bit, i.e., *b*_x_ = 1, we will thus activate the gate *X* in Fig. 5, therefore, the state of Eq. (5f) will be affected as follows:

7 $$\begin{gathered} \left| {\psi \left( {t_{5}^{\prime} } \right)} \right\rangle = \left( {X \otimes I \otimes I \otimes I} \right)\left| {\psi \left( {t_{5} } \right)} \right\rangle \hfill \\ \quad \quad \quad = \left( {\left[ {\begin{array}{*{20}c} 0 & {\quad 1} \\ 1 & {\quad 0} \\ \end{array} } \right] \otimes \left[ {\begin{array}{*{20}c} 1 & {\quad 0} \\ 0 & {\quad 1} \\ \end{array} } \right] \otimes \left[ {\begin{array}{*{20}c} 1 & {\quad 0} \\ 0 & {\quad 1} \\ \end{array} } \right] \otimes \left[ {\begin{array}{*{20}c} 1 & {\quad 0} \\ 0 & {\quad 1} \\ \end{array} } \right]} \right)\left| {\psi \left( {t_{5} } \right)} \right\rangle \hfill \\ \quad \quad \quad = \left[ {\begin{array}{*{20}c} {\begin{array}{*{20}c} {\begin{array}{*{20}c} {\begin{array}{*{20}c} {\begin{array}{*{20}c} 0 & {\quad 1} & {\quad 0} & {\quad 0} \\ \end{array} } & {\quad 0} & {\quad 0} & {\quad 0} \\ \end{array} } & {\quad 0} & {\quad 1} & {\quad 0} \\ \end{array} } & {\quad 0} & {\quad 0} & {\quad 0} \\ \end{array} } & {\quad 0} & {\quad 0} & {\quad 0} \\ \end{array} } \right]^{T} {{\sqrt 2 } \mathord{\left/ {\vphantom {{\sqrt 2 } 4}} \right. \kern-0pt} 4}. \hfill \\ \end{gathered}$$

If we now apply to Eq. (7) the same procedure that concluded in Eq. (6), we will have:

8 $$\begin{gathered} \left| {\psi \left( {t_{6}^{\prime} } \right)} \right\rangle = \left( {I \otimes I \otimes SWAP} \right)\left( {I \otimes SWAP \otimes I} \right)\left| {\psi \left( {t_{5}^{\prime} } \right)} \right\rangle \hfill \\ \quad \quad \quad = \left( {\left[ {\begin{array}{*{20}c} 1 & {\quad 0} \\ 0 & {\quad 1} \\ \end{array} } \right] \otimes \left[ {\begin{array}{*{20}c} 1 & {\quad 0} \\ 0 & {\quad 1} \\ \end{array} } \right] \otimes \left[ {\begin{array}{*{20}c} 1 & {\quad 0} & {\quad 0} & {\quad 0} \\ 0 & {\quad 0} & {\quad 1} & {\quad 0} \\ 0 & {\quad 1} & {\quad 0} & {\quad 0} \\ 0 & {\quad 0} & {\quad 0} & {\quad 1} \\ \end{array} } \right]} \right)\left( {\left[ {\begin{array}{*{20}c} 1 & {\quad 0} \\ 0 & {\quad 1} \\ \end{array} } \right] \otimes \left[ {\begin{array}{*{20}c} 1 & {\quad 0} & {\quad 0} & {\quad 0} \\ 0 & {\quad 0} & {\quad 1} & {\quad 0} \\ 0 & {\quad 1} & {\quad 0} & {\quad 0} \\ 0 & {\quad 0} & {\quad 0} & {\quad 1} \\ \end{array} } \right] \otimes \left[ {\begin{array}{*{20}c} 1 & {\quad 0} \\ 0 & {\quad 1} \\ \end{array} } \right]} \right)\left| {\psi \left( {t_{5}^{\prime} } \right)} \right\rangle \hfill \\ \quad \quad \quad = \left[ {\begin{array}{*{20}c} {\begin{array}{*{20}c} {\begin{array}{*{20}c} {\begin{array}{*{20}c} {\begin{array}{*{20}c} 0 & 0 & 0 & 0 \\ \end{array} } & 1 & 0 & 0 \\ \end{array} } & 0 & 1 & 0 \\ \end{array} } & 0 & 0 & 0 \\ \end{array} } & 0 & 0 & 0 \\ \end{array} } \right]^{T} {{\sqrt 2 } \mathord{\left/ {\vphantom {{\sqrt 2 } 4}} \right. \kern-0pt} 4} \hfill \\ \quad \quad \quad = \left| {\beta_{01} } \right\rangle \left| {00} \right\rangle {{\sqrt 2 } \mathord{\left/ {\vphantom {{\sqrt 2 } 4}} \right. \kern-0pt} 4}. \hfill \\ \end{gathered}$$

Equations (6) and (8) constitute a double theoretical verification that the new protocol works correctly, given that the following can be directly observed:

1. at the output of Fig. 3 we effectively have a shared state of type $\left| {\beta_{00} } \right\rangle$ between the qubits q[0] and q[3] and as a consequence,
2. by applying the *X* gate to the qubit q[0], a new Bell state is shared between the qubits q[0] and q[3], where in this case it is a $\left| {\beta_{01} } \right\rangle$ state.

**Figure 6:**
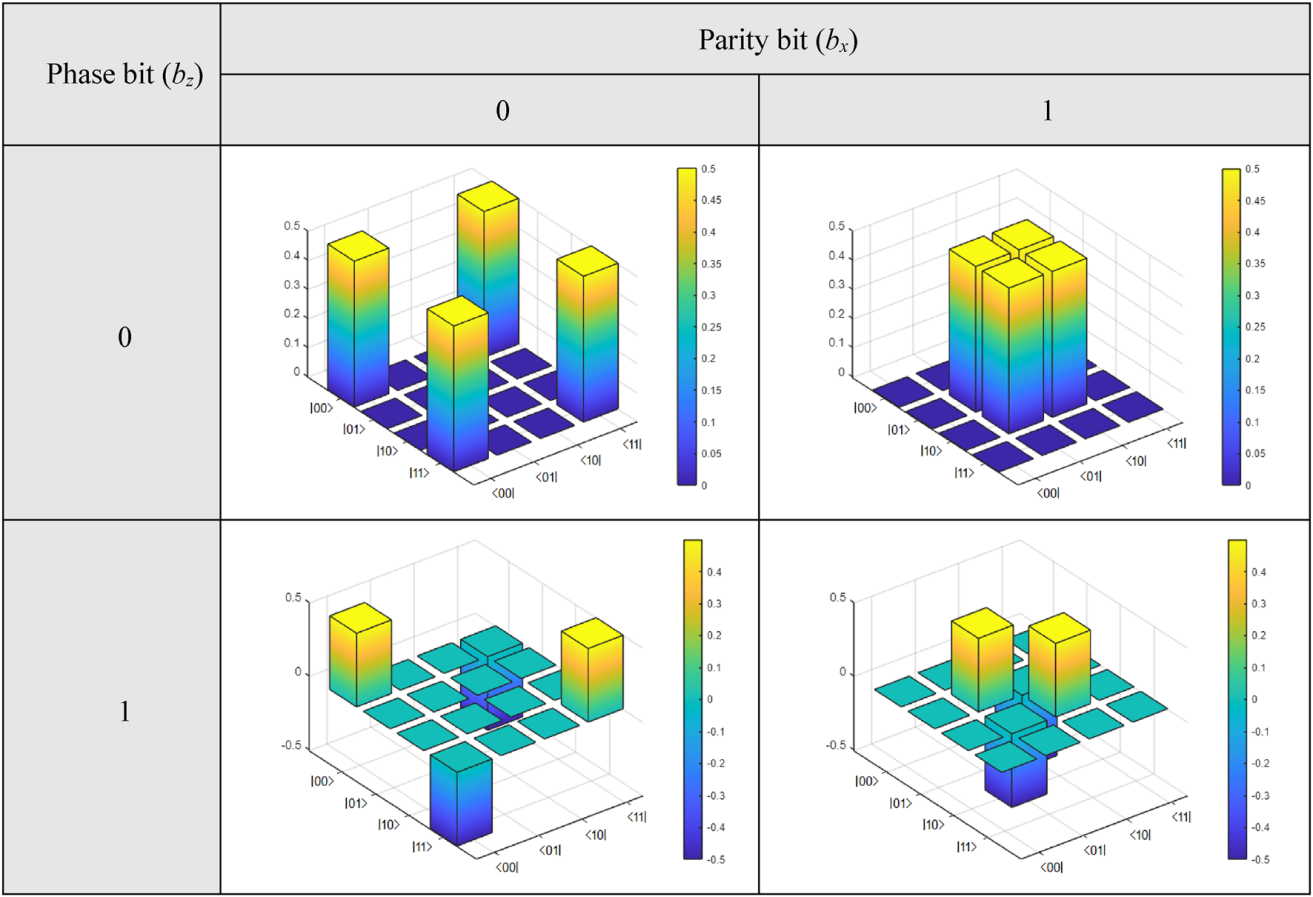
$\left| {{\upbeta }_{{b_{z} b_{x} }} } \right\rangle$ states density matrices in terms of phase and parity bits.

Finally, Fig. 6 shows the theoretical 3D density matrices of the Bell states in terms of phase and parity bits {*b*_x_, *b*_z_}. During the implementation of this experiment on the optical table, we will compare these matrices with those obtained there. As we have mentioned before, in that case, both Pauli matrices {*X*, *Z*} will be implemented by adjustments to the angles *θ* of two half-wave plates (HWPs), where said angle will be *θ* = {0, π/4} ⇌ {*X*, *Z*}, respectively.

## Bell test from two independent sources of entangled photons

**Figure 7:**
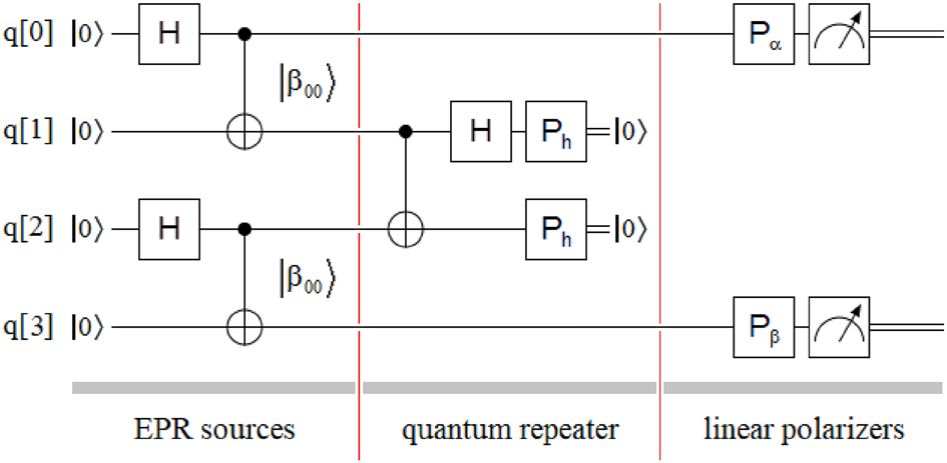
Bell test from two independent sources of entangled photons: one linear polarizer *P*_α_ in q[0], and other *P*_β_ in q[3] allow to complete the configuration to evaluate the coincidences based on angles *α* and *β*.

Except for the fact that the Bell state of the $\left| {\beta_{00} } \right\rangle$-type, shared between the qubits q[0] and q[3], results from the application of the presented entanglement swapping protocol, given that before this these qubits were completely independent, the experiment Fig. 7 represents a simple Bell test^78–81^. In this way, if the protocol works correctly and the qubits q[0] and q[3] really share a Bell state of the $\left| {\beta_{00} } \right\rangle$- type, the appropriate rotation of the linear polarizers, *P*_α_ in the qubit q[0] and *P*_β_ in the qubit q[3], they should show us the probability curves that are typical in this type of experiment. Then, defining the 2D rotation matrix for a generic angle *θ* as:

9 $$R_{\theta } = \left[ {\begin{array}{*{20}c} {\cos \left( \theta \right)} & {\quad - \sin \left( \theta \right)} \\ {\sin \left( \theta \right)} & {\quad \cos \left( \theta \right)} \\ \end{array} } \right]$$

we can define the generic polarizer *P*_θ_ from *R*_θ_,

10 $$P_{\theta } = R_{\theta } P_{h} R_{\theta }^{T}$$

Therefore, if we now define a generic outcome because of the application of the generic polarizer *P*_θ_, it results:

11 $$O_{\theta } = \left[ {\begin{array}{*{20}c} {\cos \left( \theta \right)} \\ {\sin \left( \theta \right)} \\ \end{array} } \right]$$

Consequently, for the case of Fig. 7, in which we have a Bell state $\left| {\beta_{00} } \right\rangle$ shared between the qubits q[0] and q[3], and a polarizer *P*_α_ in the qubit q[0] and a polarizer *P*_β_ in the qubit q[3], the associated probability is:

12a $$P_{00} = \left\langle {O_{\alpha } \otimes O_{b} } \right|\left. {\beta_{00} } \right\rangle^{2} = {\raise0.7ex\hbox{$1$} \!\mathord{\left/ {\vphantom {1 2}}\right.\kern-0pt} \!\lower0.7ex\hbox{$2$}}\cos \left( {\alpha - \beta } \right)^{2}$$

The result obtained in Eq. (12a), corresponds to the case that before the polarizer *P*_α_, we do not activate any of the *X* or *Z* matrices. If instead, we decide to activate Pauli gates, as in the case of Fig. 5, for each remaining Bell state we will have:

12b $$P_{01} = \left\langle {O_{\alpha } \otimes O_{b} } \right|\left. {\beta_{01} } \right\rangle^{2} = {\raise0.7ex\hbox{$1$} \!\mathord{\left/ {\vphantom {1 2}}\right.\kern-0pt} \!\lower0.7ex\hbox{$2$}}\sin \left( {\alpha + \beta } \right)^{2}$$

12c $$P_{10} = \left\langle {O_{\alpha } \otimes O_{b} } \right|\left. {\beta_{10} } \right\rangle^{2} = {\raise0.7ex\hbox{$1$} \!\mathord{\left/ {\vphantom {1 2}}\right.\kern-0pt} \!\lower0.7ex\hbox{$2$}}\cos \left( {\alpha + \beta } \right)^{2}$$

12d $$P_{11} = \left\langle {O_{\alpha } \otimes O_{b} } \right|\left. {\beta_{11} } \right\rangle^{2} = {\raise0.7ex\hbox{$1$} \!\mathord{\left/ {\vphantom {1 2}}\right.\kern-0pt} \!\lower0.7ex\hbox{$2$}}\sin \left( {\alpha - \beta } \right)^{2}$$

In the experiment on the optical table, we will evaluate the performance of Fig. 7 when we work with a Bell state of type $\left| {\beta_{00} } \right\rangle$ between the qubits q[0] and q[3]. In this case, we assign a fixed angle to *P*_β_ from the set *β* = {0, π/4, π/2, 3π/4}, and for each of those angles, we rotate the polarizer *P*_α_ completely. If the new entanglement swapping protocol works correctly, we should experimentally verify the coincidences or probabilities of Eq. (12a).

## Optical table

Next, we will implement the experiments from the previous section on the optical table, for which this section is organized into three well-defined subsections:

- Preparation of the configuration common to both experiments,
- Bell state change test, and
- Bell test from two independent sources of entangled photons.

*Preparation of the configuration common to both experiments*.- Here, we will carry out the linear tomography^82^ and the maximum likelihood tomography^82^ from the experimental data of the two EPR sources of the $\left| {\beta_{00} } \right\rangle$-type used. With these, we calculate the respective density matrices of both sources. The density matrix from the linear tomography of Source #1 (LT1) is:

13a $$\rho_{{{\text{LT}}1}} = \left[ {\begin{array}{*{20}l} {{0}{\text{.4858}}} \hfill & {\quad - {0}{\text{.0054 + 0}}{\text{.0114i}}} \hfill & {\quad - {0}{\text{.0094}} - {0}{\text{.0178i}}} \hfill & {\quad {0}{\text{.5182 + 0}}{\text{.0380i}}} \hfill \\ { - {0}{\text{.0054}} - {0}{\text{.0114i}}} \hfill & {\quad {0}{\text{.0042}}} \hfill & {\quad {0}{\text{.0260}} - {0}{\text{.0146i}}} \hfill & {\quad - {0}{\text{.0656}} - {0}{\text{.0076i}}} \hfill \\ { - {0}{\text{.0094 + 0}}{\text{.0178i}}} \hfill & {\quad {0}{\text{.0260 + 0}}{\text{.0146i}}} \hfill & {\quad {0}{\text{.0057}}} \hfill & {\quad - {0}{\text{.0690 + 0}}{\text{.0134i}}} \hfill \\ {{0}{\text{.5182}} - {0}{\text{.0380i}}} \hfill & {\quad - {0}{\text{.0656 + 0}}{\text{.0076i}}} \hfill & {\quad - {0}{\text{.0690}} - {0}{\text{.0134i}}} \hfill & {\quad {0}{\text{.5011}}} \hfill \\ \end{array} } \right]$$

while the density matrix from the maximum likelihood tomography of Source #1 (MT1) is:

13b $$\rho_{{{\text{MT}}1}} = \left[ {\begin{array}{*{20}l} {{0}{\text{.5055}}} \hfill & {\quad - {0}{\text{.0251 + 0}}{\text{.0106i}}} \hfill & {\quad - {0}{\text{.0408}} - {0}{\text{.0221i}}} \hfill & {\quad {0}{\text{.4823 + 0}}{\text{.0329i}}} \hfill \\ { - {0}{\text{.0251}} - {0}{\text{.0106i}}} \hfill & {\quad {0}{\text{.0045}}} \hfill & {\quad {0}{\text{.0012 + 0}}{\text{.0019i}}} \hfill & {\quad - {0}{\text{.0304}} - {0}{\text{.0077i}}} \hfill \\ { - {0}{\text{.0408 + 0}}{\text{.0221i}}} \hfill & {\quad {0}{\text{.0012}} - {0}{\text{.0019i}}} \hfill & {\quad {0}{\text{.0040}}} \hfill & {\quad - {0}{\text{.0420 + 0}}{\text{.0192i}}} \hfill \\ {{0}{\text{.4823}} - {0}{\text{.0329i}}} \hfill & {\quad - {0}{\text{.0304 + 0}}{\text{.0077i}}} \hfill & {\quad - {0}{\text{.0420}} - {0}{\text{.0192i}}} \hfill & {\quad {0}{\text{.4830}}} \hfill \\ \end{array} } \right]$$

The density matrix from the linear tomography of Source #2 (LT2) is:

13c $$\rho_{{{\text{LT}}2}} = \left[ {\begin{array}{*{20}l} {{0}{\text{.4885}}} \hfill & {\quad - {0}{\text{.0039 + 0}}{\text{.0114i}}} \hfill & {\quad - {0}{\text{.0085}} - {0}{\text{.0178i}}} \hfill & {\quad {0}{\text{.5189 + 0}}{\text{.0380i}}} \hfill \\ { - {0}{\text{.0039}} - {0}{\text{.0114i}}} \hfill & {\quad {0}{\text{.0029}}} \hfill & {\quad {0}{\text{.0261}} - {0}{\text{.0146i}}} \hfill & {\quad - {0}{\text{.0655}} - {0}{\text{.0076i}}} \hfill \\ { - {0}{\text{.0085 + 0}}{\text{.0178i}}} \hfill & {\quad {0}{\text{.0261 + 0}}{\text{.0146i}}} \hfill & {\quad {0}{\text{.0066}}} \hfill & {\quad - {0}{\text{.0709 + 0}}{\text{.0134i}}} \hfill \\ {{0}{\text{.5189}} - {0}{\text{.0380i}}} \hfill & {\quad - {0}{\text{.0655 + 0}}{\text{.0076i}}} \hfill & {\quad - {0}{\text{.0709}} - {0}{\text{.0134i}}} \hfill & {\quad {0}{\text{.5007}}} \hfill \\ \end{array} } \right]$$

while the density matrix from the maximum likelihood tomography of Source #2 (MT2) is:

13d $$\rho_{{{\text{MT}}2}} = \left[ {\begin{array}{*{20}l} {{0}{\text{.5082}}} \hfill & {\quad - {0}{\text{.0236 + 0}}{\text{.0106i}}} \hfill & {\quad - {0}{\text{.0399}} - {0}{\text{.0221i}}} \hfill & {\quad {0}{\text{.4830 + 0}}{\text{.0329i}}} \hfill \\ { - {0}{\text{.0236}} - {0}{\text{.0106i}}} \hfill & {\quad {0}{\text{.0032}}} \hfill & {\quad {0}{\text{.0013 + 0}}{\text{.0019i}}} \hfill & {\quad - {0}{\text{.0303}} - {0}{\text{.0077i}}} \hfill \\ { - {0}{\text{.0399 + 0}}{\text{.0221i}}} \hfill & {\quad {0}{\text{.0013}} - {0}{\text{.0019i}}} \hfill & {\quad {0}{\text{.0049}}} \hfill & {\quad - {0}{\text{.0439 + 0}}{\text{.0192i}}} \hfill \\ {{0}{\text{.4830}} - {0}{\text{.0329i}}} \hfill & {\quad - {0}{\text{.0303 + 0}}{\text{.0077i}}} \hfill & {\quad - {0}{\text{.0439}} - {0}{\text{.0192i}}} \hfill & {\quad {0}{\text{.4826}}} \hfill \\ \end{array} } \right]$$

**Figure 8:**
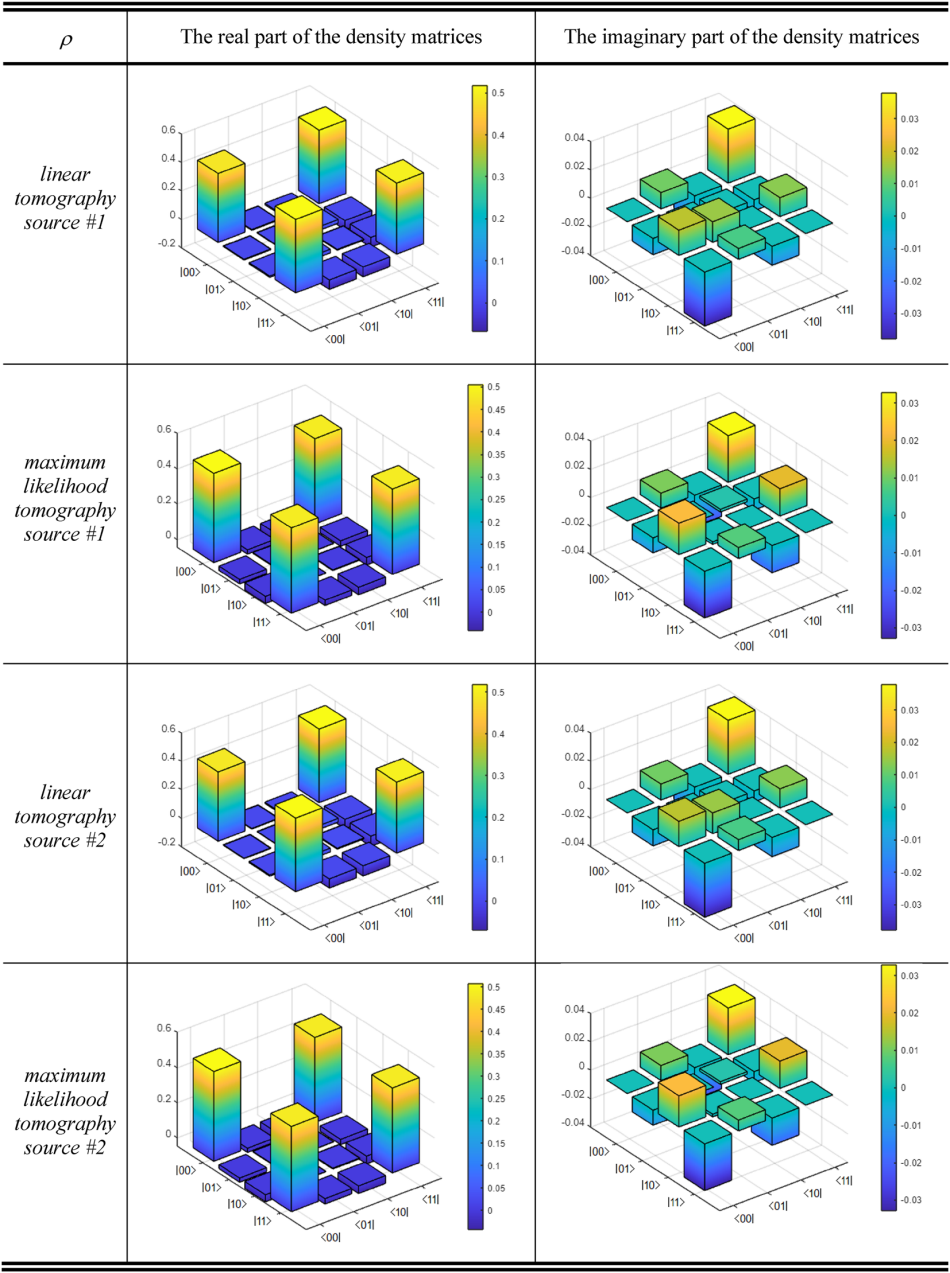
Density matrices from linear and maximum likelihood tomographies for both sources.

The four density matrices of Eq. (13) can be seen in Fig. 8. From these density matrices, we will obtain the tangle, the linear entropy, the Fidelity, and the errors. The state was calibrated several times to progressively improve the tangle. Two completely independent sources of entangled photons were used, from two 405nm pump lasers, with a range of power between 20mw and 50mW, and two pairs of solid blocks (5 × 5 × 3 mm^3^) of β-barium-borate (BBO) crystal to produce Type-I down-conversion. In each source, one crystal was attached after another, where the second is rotated 90º with respect to the first. Although in each source, two Type-I crystals were used, the joint action of both crystals mutually rotated by 90º generates Bell states of the $\left| {\beta_{00} } \right\rangle$-type.

We use two identical gallium-nitride (GaN) diode lasers for two reasons:

- it has greater stability and temperature control, and.
- its short wavelength allows us to work with efficient detectors of 810 nm.

Each blue diode laser beam (405 nm, 50mW) passes through a zero-order half-wave plate (HWP) with a phase of 22.5º, which represents a Hadamard matrix^6^. Then, the laser beam passes through a narrow bandpass filter or quartz plate of 405 nm, i.e., a ∝ -BBO, as a phase-matching crystal. Thanks to this, photons with a state of polarization of diagonal type $\left| + \right\rangle$ are obtained. At each source, a beam with that polarization enters the first crystal, it generates a pair $\left| {HH} \right\rangle$, while upon entering the second crystal, it generates a pair $\left| {VV} \right\rangle$, in such a way that together both pairs generate the state $\left| {\beta_{00} } \right\rangle$.

After each configuration of double Type-I crystal, a BBO is used on each beam. Besides, a block of pinhole + filter(808 nm) + lens at the entrance of each avalanche photodiode (APD). Other elements were used in the compensation and calibration phases.

Using a 405 nm laser pump, this configuration produces a 6º cone at the output of the second crystal, i.e., 3º for the branch known as signal (810 nm) and -3º for the branch known as idler (810 nm), with a phase matching angle for Type-I down-conversion of approximately 29º. In both figures, 9 and 11, the angle between both beams (signal and idler) was exaggerated to better appreciate the layout, where the beam path dimensions are not to scale.

**Figure 9:**
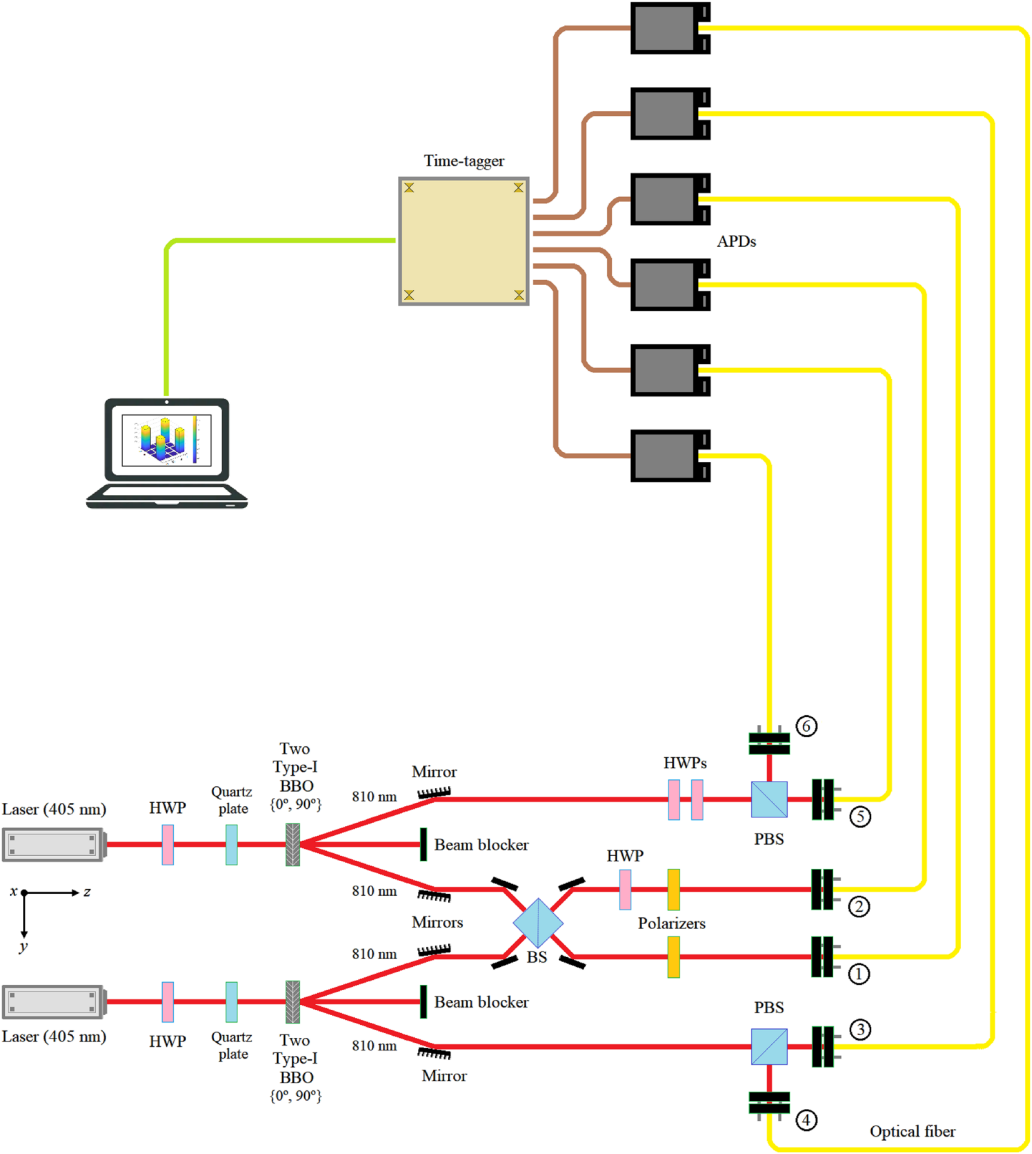
Bell state change test on the optical table. The angles of the HWPs located in the upper beam are set manually, such that {0, π/4} ⇌ {*X*, *Z*}, respectively. The results obtained by all the detectors, from 1 to 6, are sent to their respective APDs, and from these to a time-tagger, which collects all the signals coming from the APDs at the same time and sends them to the computer to obtain the final density matrices of the four Bell states obtained.

Continuing with the implementation of the protocols of Figs. 9 and 11, both outgoing beams of the second BBO are deflected by two mirrors so that all the geometry of the protocol is completely contained within the useful perimeter of the optical table. A third beam collinear with the beam incident to the first BBO crystal and forming the same angle with respect to the two beams mentioned before (signal, and idler) is intercepted by a beam blocker.

Two dual-wavelength 405/810 nm polarizing beam-splitter (PBS) of 0.5″ × 0.5″ × 0.5″ are used in both experiments. When working at 810 nm, the APDs have an efficiency of 60%, and we have worked with acquisition times ranging from 50 ms to 1 s, with and without a block of pinhole + filter(808 nm) + lens before the APDs. A six-channel time-tagger device is used after the four APDs.

Several elements are not shown in Figs. 9 and 11 of both experiments on the optical table so as not to complicate them.

In both experiments, the quantum repeater is implemented thanks to a beam-splitter of 808/810 nm, four mirrors, a zero-order half-wave plate (HWP) with a phase of 22.5º, which represents a Hadamard matrix^6^, and two 810 nm calcite film polarizer fixed at horizontal polarization (which is good throughout the visible spectrum and has high extinction ratios).

Two lasers were used although a single one could have been used and resorted to the central outgoing beam of the spontaneous parametric down conversion (SPDC) process^77^ at the exit of the first BBO crystal, so that it impacts a new pair of Type I crystals and thus generate the second pairs of entangled photons^83^, but we abandoned this option to avoid any eventual spurious coupling between the pairs of entangled photons from both sources that would disturb the performance of the experiments. In this way, aligning both independent configurations to produce entangled photons of the $\left| {\beta_{00} } \right\rangle$-type, the metrics obtained with each source were the following:

- *Source #1:* tangle = 0.9715 ± 0.0003, linear entropy = 0.0648, Fidelity = 0.9819 ± 0.0001, and errors of the order of 0.014, and
- *Source #2:* tangle = 0.9685 ± 0.0003, linear entropy = 0.0703, Fidelity = 0.9783 ± 0.0001, and errors of the order of 0.014.

As we can see, both sources have practically identical metrics, which is extremely convenient for the performance of both experiments.

*Bell state change test*.-Fig. 9 shows the layout of this experiment, with two 808/810nm zero-order half-wave plates (HWP) employed for the implementation of the *X* and *Z* Pauli matrices with angles of {0, π/4} ⇌ {*X*, *Z*}, respectively. The intervention of these HWPs, as well as their values depends on the transition that is carried out between the original state generated by each source of entangled photons, i.e., $\left| {\beta_{00} } \right\rangle$, and the states that must be reached to verify the performance of the new protocol and that are the central axis of the present experiment, that is, $\left\{ {\left| {\beta_{01} } \right\rangle ,\left| {\beta_{10} } \right\rangle ,\left| {\beta_{11} } \right\rangle } \right\}$. In this way, these transitions are:

14a $${\text{I}} \times {\text{HWP}}\left( {{\pi \mathord{\left/ {\vphantom {\pi 4}} \right. \kern-0pt} 4}} \right) = IX = X \Rightarrow \left| {\beta_{00} } \right\rangle \to \left| {\beta_{01} } \right\rangle ,$$

14b $${\text{HWP}}\left( 0 \right) \times {\text{I}} = ZI = Z \Rightarrow \left| {\beta_{00} } \right\rangle \to \left| {\beta_{10} } \right\rangle ,\;{\text{and}}$$

14c $${\text{HWP}}\left( 0 \right) \times {\text{HWP}}\left( {{\pi \mathord{\left/ {\vphantom {\pi 4}} \right. \kern-0pt} 4}} \right) = ZX \Rightarrow \left| {\beta_{00} } \right\rangle \to \left| {\beta_{11} } \right\rangle .$$

In Eq. (14), the identity matrix *I* represents the absence of an HWP in that position. The angles of both HWPs located in the upper beam of Fig. 9 are set manually, according to the transitions of Eq. (14). The results obtained by the six detectors, labeled from 1 to 6, are sent to their respective APDs, and from these to a time-tagger, which collects all the signals coming from the APDs at the same time and sends them to the computer to obtain the final density matrices of the four Bell states obtained.

**Figure 10:**
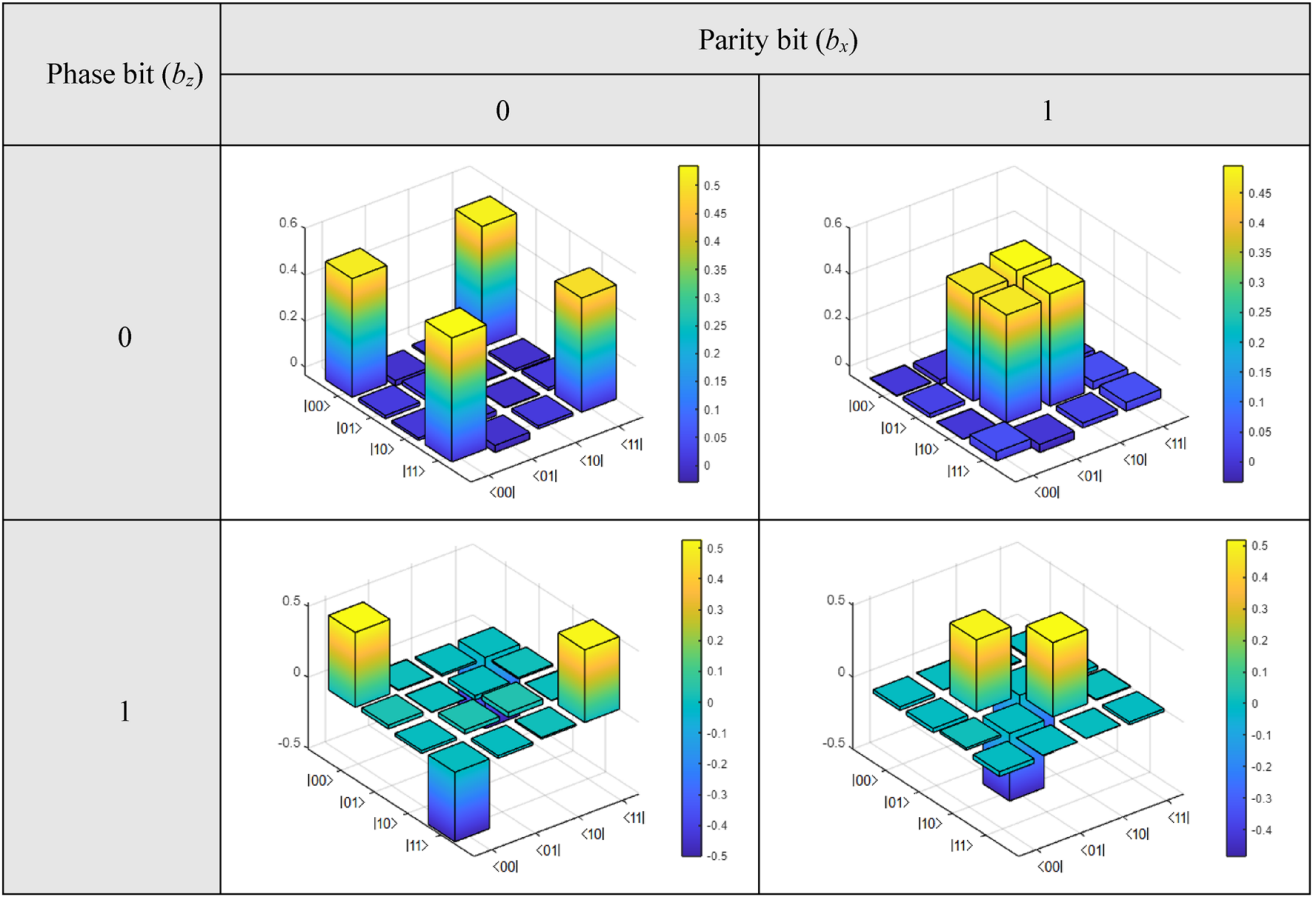
$\left| {{\upbeta }_{{b_{z} b_{x} }} } \right\rangle$ states density matrices in terms of phase and parity bits.

Figure 10 shows the state density matrices in terms of the phase and the parity bits, both for the original state $\left| {\beta_{00} } \right\rangle$ generated because of the action of the new protocol and those due to the transitions resulting from Eq. (14). In Fig. 5, those bits acted as exponents of the respective Pauli matrices {*Z, X*}, therefore,

the parity and phase bits of Fig. 10 act as activation bits {*b*_z_, *b*_x_} of the HWPs that represent the Pauli matrices *X* and *Z*, giving rise to the transitions of Eq. (14), so that, for {*b*_z_, *b*_x_} = {0, 0}, we see the density matrix corresponding to the Bell state generated by the new protocol, i.e., $\left| {\beta_{00} } \right\rangle$, while for the remaining cases, we have {*b*_z_, *b*_x_} = {0, 1} =  > $\left| {\beta_{01} } \right\rangle$, {*b*_z_, *b*_x_} = {1, 0} =  > $\left| {\beta_{10} } \right\rangle$, and {*b*_z_, *b*_x_} = {1, 1} =  > $\left| {\beta_{11} } \right\rangle$.

A simple visual inspection shows the similarity between Figs. 6 and 10, theoretical and experimental, respectively, which tells us about the excellent performance of the new protocol, leaving no doubt that the extreme qubits of the protocol share a common $\left| {\beta_{00} } \right\rangle$-type entangled state.

**Figure 11:**
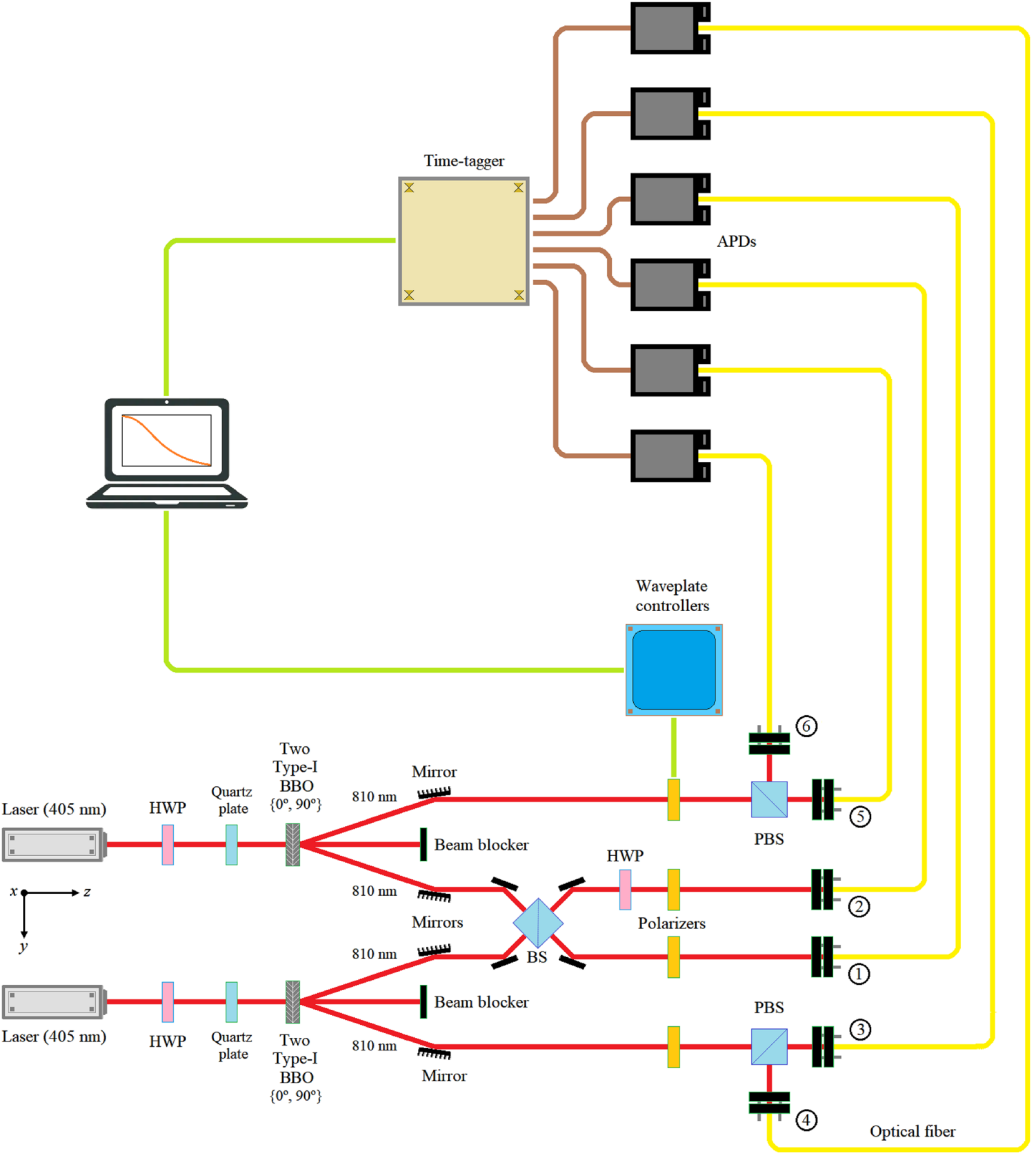
Bell test from two independent sources of entangled photons on the optical table. Taking into account everything common with the experiment in Fig. 9, this experiment consists of two polarizers interrupting the upper and lower beam of the figure, one fixed, the lower one, at each of the angles *β* = {0, π/4, π/2, 3π/4}, while the other varies according to the commands that the computer sends to the waveplate controller, responsible for the variation of the angle in the upper polarizer.

*Bell test from two independent sources of entangled photons*.-As we can see in Fig. 11, at the end of the signal beam of Source #1, we interrupt the beam with the polarizer *P*_α_, while we do the same with Source #2, in its idler beam, with the polarizer *P*_β_. The *P*_β_ polarizer, an 810nm calcite film polarizer, is set manually, while the *P*_α_ polarizer can be implemented using some of the following configurations:

- an 808/810 nm rotatable polarizer (*P*_θ_), i.e., a unique and special variable-angle-device, where the angle of the polarizer is controlled using external commands coming from the waveplate controller,
- two 808 nm electro-optical modulators (EOM): $EOM\left( \theta \right) \times P_{h} \times EOM\left( \theta \right)$, and
- two 808/810 nm HWPs: $HWP\left( {{\theta \mathord{\left/ {\vphantom {\theta 2}} \right. \kern-0pt} 2}} \right) \times P_{h} \times HWP\left( {{\theta \mathord{\left/ {\vphantom {\theta 2}} \right. \kern-0pt} 2}} \right)$.

In cases (b and c), the angle is also controlled by external commands coming from the waveplate controller. For this experiment, option (b) was used due to the availability of its components in our laboratory.

As we have mentioned previously, the experiment consists of manually setting the value of the polarizer *P*_β_ at each of the following angles *β* = {0, π/4, π/2, 3π/4} and having the computer generate the necessary commands for the configuration of the option (b), corresponding a *P*_α_, sweep all the angles from 0 to 2π to plot the probability/coincidence curves of Eq. (12a). In other words, we leave the polarizer *P*_β_ fixed at one of the following angles *β* = {0, π/4, π/2, 3π/4} while *P*_α_ makes a complete rotation controlled by the waveplate controller. In this way, we will carry out a complete plot of the curves of Eq. (12a) for the four angles of the polarizer *P*_β_.

If we now make θ = α—β, and define the following coincidence rates:

- *R*(θ) is the coincidence rate in terms of the angle with both polarizers,
- *R*o is the coincidence rate with the two polarizers removed.
- *R*(θ)/*R*o is the normalized coincidence rate as a function of the relative polarizer orientation.

**Figure 12:**
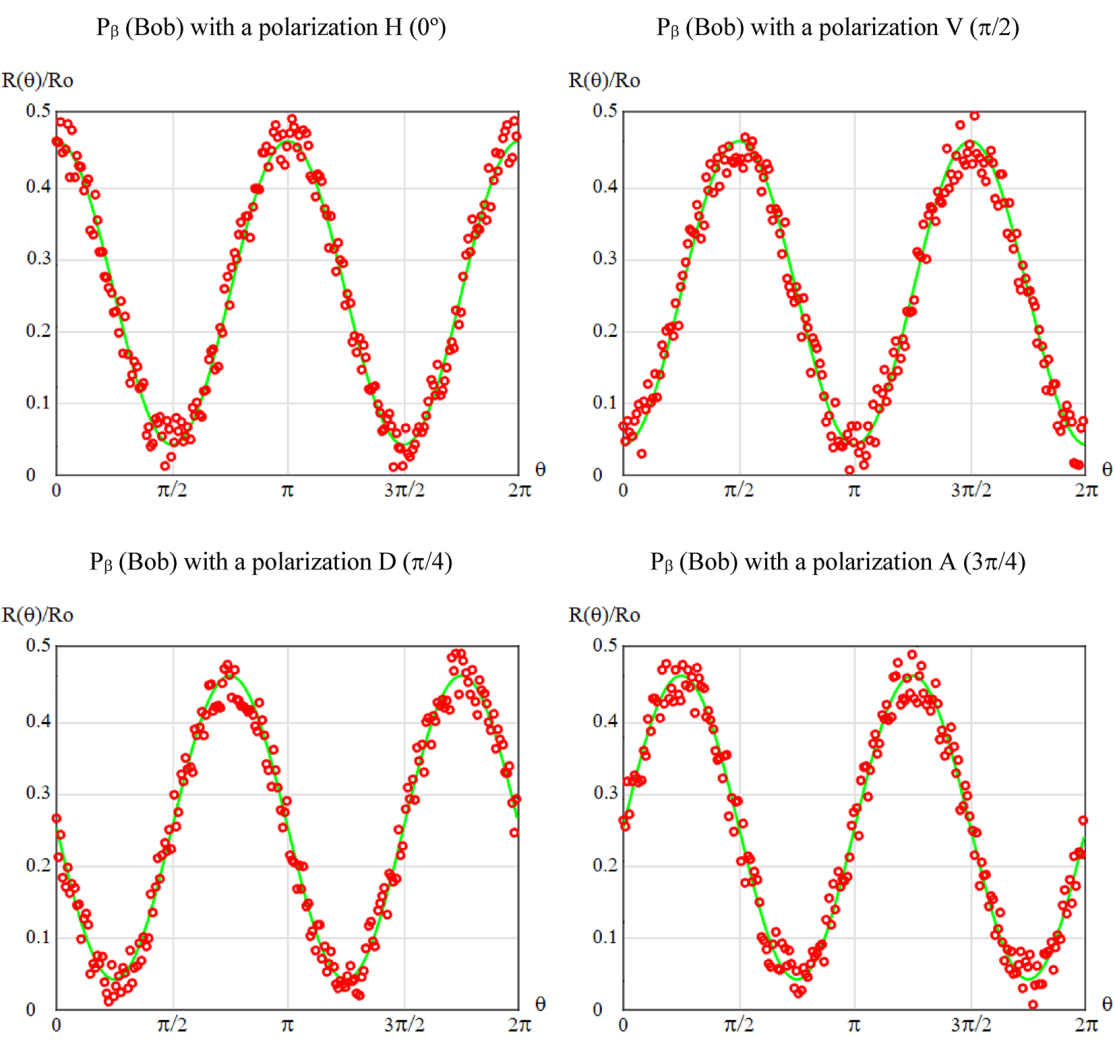
R(θ)/Ro in terms of *θ*, where *β* = {0, π/4, π/2, 3π/4} ⇌ *P*_β_ = {H, V, D, A}, respectively.

Figure 12 shows the normalized coincidence rate *R*(θ)/*R*o as a function of the relative polarizer orientation. Therefore, If Bob's polarizer *P*_β_ is set to a polarization *H* (0º), and Alice's polarizer *P*_α_ is fully and automatically rotated by the waveplate controller, it can be seen in the top-left curve of Fig. 12 that the coincidences start at a maximum since both Bob and Alice are with their horizontal polarizers. The maxima and minima alternate every π/2 rad on the graph scale). The accounts of this experiment are complementary to those of the following experiment. The curve in green represents the ideal or theoretical case of Eq. (12a), while the distribution of the red rings represents the curve that results from the coincidences found by the computer. Now, Bob's polarizer *P*_β_ is set to a polarization *V* (π/2), and Alice's polarizer *P*_α_ is again fully and automatically rotated by the waveplate controller, it can be seen in the top-right curve of Fig. 12 that the coincidences start at a minimum, since Alice always starts with horizontal polarization. In this case, the oscillations are also consistent, changing from minimum to maximum and vice versa every π/2 rad. Setting Bob *P*_β_'s polarizer to diagonal polarization *D* (π/4) or $$\left| + \right\rangle = \left[ {\begin{array}{*{20}c} {{1 \mathord{\left/ {\vphantom {1 {\sqrt 2 }}} \right. \kern-0pt} {\sqrt 2 }}} & {{1 \mathord{\left/ {\vphantom {1 {\sqrt 2 }}} \right. \kern-0pt} {\sqrt 2 }}} \\ \end{array} } \right]^{T}$$, and always carrying out the same procedure in the Alice *P*_α_ polarizer, the lower-left curve of Fig. 12 emerges, which we observe starts with an intermediate value. Something similar happens when Bob *P*_β_'s polarizer is set to anti-diagonal polarization *A* (3π/4) or $$\left| - \right\rangle = \left[ {\begin{array}{*{20}c} {{1 \mathord{\left/ {\vphantom {1 {\sqrt 2 }}} \right. \kern-0pt} {\sqrt 2 }}} & {{{ - 1} \mathord{\left/ {\vphantom {{ - 1} {\sqrt 2 }}} \right. \kern-0pt} {\sqrt 2 }}} \\ \end{array} } \right]^{T}$$, the complete rotation of Alice *P*_α_'s polarizer, always starting from the horizontal polarization, gives rise to the lower-right curve of Fig. 12, which also coincides perfectly with the expected results for this type of experiment.

Finally, the results of Fig. 12 are remarkably coincident with the theoretical probability expressed in Eq. (12a), which again proves the performance of the new protocol, given that if it did not carry out a true entanglement swapping process, it would be impossible to achieve the results of both experiments presented here.

## Discussions

**Figure 13:**
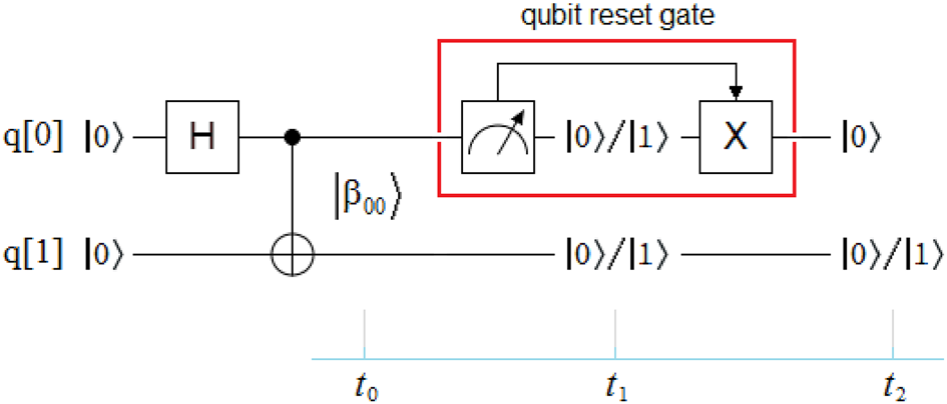
Qubit reset gate in an entanglement context. A version of the horizontal polarizer *P*_h_ in any physical machine of the IBM Q Experience program^84^, and the Rigetti Computing family^85^. Within the qubit reset gate located in the qubit q[0] at the output of the entangled pair source, the quantum measurement module generates at its output at time *t*_1_ an equiprobable result of type $$\left\{ {\begin{array}{*{20}c} {\left| 0 \right\rangle } \\ {50\% } \\ \end{array} \begin{array}{*{20}c} , \\ {} \\ \end{array} \begin{array}{*{20}c} {\left| 1 \right\rangle } \\ {50\% } \\ \end{array} \;} \right\}$$. This same result is projected to its partner qubit q[1]. If the output of the quantum measurement module is $\left| 0 \right\rangle$, the inverse *X* is not activated, but if the output is $\left| 1 \right\rangle$, then the inverter *X* is activated, so that the output of the qubit reset gate is always $\left| 0 \right\rangle$ at time *t*_2_. However, the qubit q[1] will end up with any of the two possible values, thus not generating the result necessary for the correct functioning of the protocol according to Eq. (5).

As we could observe throughout this study, the new protocol requires the intensive use of horizontal polarizers, such as those mentioned in Eq. (3). For this reason, this protocol can only be implemented optically, given that the version of the horizontal polarizers used in the physical computers of the IBM Q Experience program^84^, as well as those of Rigetti Computing^85^, called qubit reset gate, are of the type shown in Fig. 13. The problem with this implementation is the following: within the qubit reset gate highlighted in red and located in the qubit q[0] at the output of the entangled pair source of the $\left| {\beta_{00} } \right\rangle$-type, the quantum measurement module generates at its output at time *t*_1_ an equiprobable result of type $$\left\{ {\begin{array}{*{20}c} {\left| 0 \right\rangle } \\ {50\% } \\ \end{array} \begin{array}{*{20}c} , \\ {} \\ \end{array} \begin{array}{*{20}c} {\left| 1 \right\rangle } \\ {50\% } \\ \end{array} \;} \right\}$$. This same result is projected to its partner qubit q[1] at that same moment. If the output of the quantum measurement module turns out to be $\left| 0 \right\rangle$, the inverter Pauli matrix *X* is not activated, but if the output turns out to be $\left| 1 \right\rangle$, then the inverter *X* is activated, so that the output of the qubit reset gate is always $\left| 0 \right\rangle$ at time *t*_2_. However, the qubit q[1] will end up with any of the two possible values, that is to say, the result necessary for the correct functioning of the protocol according to Eq. (5) is never generated. This way of implementing the qubit reset gate is known as the dynamic version, which is 1000 times faster than the static version, the latter being the version required by Eq. (5) to be able to correctly implement the new protocol on any platform of the IBM Q Experience program^84^ or Rigetti Computing^85^. This is the reason why these companies do not implement a matrix version more like that of the linear polarizer used in optical implementations.

Currently, the qubit reset gate is only implemented in the manner required by Eq. (5) in simulators such as Quirk^86^, and Quantum Programming Studio^87^.

Although not necessary for the correct operation of the protocol, the 3dB drop seen in Eq. (5f) can be compensated by an optical amplifier^88^.

For the experimental verification of the performance of the proposed protocol, two remarkably different experiments were carried out, both from a theoretical point of view, according to Figs. 5 and 7, as experimental on the optical table, from Figs. 9 and 11. The most outstanding difference between both experiments lies in the static nature of the first and dynamic nature of the second since in the experiment of Fig. 9, the angles of both HWP are set in order to establish which of the gates *X* and/or Z have to interrupt the beam corresponding to the signal ray of the upper pair, then the average of all the states measured in the computer and it is confirmed that we are in the presence of another Bell state different from the original one established in $\left| {\beta_{00} } \right\rangle$ between the signal beam of the upper pair and the idler beam of the lower pair. On the other hand, in the experiment in Fig. 11, the polarizer *P*_∝_ of the signal beam of the upper pair rotates from 0º to 2π according to the orders of the waveplate controller, while the polarizer *P*_β_ of the idler beam of the lower pair remains fixed at some of them four relevant cases, i.e., {H, V, D, A}. Both experiments equally show that they could only give the results obtained in Figs. 10 and 12 if the signal beam of the upper pair shares a $\left| {\beta_{00} } \right\rangle$-type state with the idler beam of the lower pair, i.e. if the new entanglement swapping protocol has worked correctly.

Regarding the savings in implementation costs of the proposed version of the entanglement swapping protocol compared to the original, we can say that for two independent EPR sources, which implies one quantum repeater, we save one {Controlled-Z, Controlled-X} pair and two classical channels, and for four independent EPR sources, which implies three quantum repeaters, we save three {Controlled-Z, Controlled-X} pairs and six classical channels, then, for *N*-independent EPR sources, which implies *N*-1 quantum repeaters, we save *N*-1 {Controlled-Z, Controlled-X} pair and 2(*N*-1) classical channels.

The configuration of the new protocol implies more than a simple saving of physical resources during the implementation of a certain network since the proposed architecture facilitates the creation of reusable and interchangeable commercial modules in a way as natural as if they were building blocks to be used in a wall.

Finally, although this new version of the entanglement swapping protocol does not use classical disambiguation channels or unitary transforms like the original version, the protocol itself does not transmit information, but simply couples and decouples conveniently qubits from two independent sources of entangled photons, generating a third entangled state common to the most extreme qubits of the configuration to extend the range of entanglement. In this way, the range extension is more efficient when implementing a quantum network in fiber optics or free-space configurations.

## Conclusions

The possibility of eliminating multiple classical channels as well as the corresponding unitary transforms allows for dramatically lower implementation costs of the new entanglement swapping protocol compared to the original version for extensive fiber optic laying, as well as long distances between member satellites of a space quantum network, which shows a clear projection of the new protocol on the future quantum internet. Furthermore, the replacement of the classical channels and their corresponding unitary transforms with the linear horizontal polarizers significantly simplifies the implementation of the new protocol on the optical table for both experiments developed in this work to study its performance. All this translates into savings in quantum memory because the new protocol is clearly less sensitive to synchronization problems between qubits than its original counterpart.

Both experiments equally demonstrated the high performance of the new version of the protocol, which allows us to estimate a similar performance in future implementations of the new protocol when it is involved in configurations that transmit information thanks to its intervention.

The fact that this protocol can only be implemented in optical environments does not represent a limitation at all since it is the natural environment in which it should intervene.

Finally, the complete dataset with the experimental outcomes of both experiments can be acquired according to the conditions established in the *data availability statement*.

### Supplementary Information

Supplementary Information.

## Data Availability

The data that support the findings of this study are available on request from the corresponding author.
