# Supplementary material for: Simplified entanglement swapping protocol for the quantum Internet
Source: Sci Rep. 2023 Dec 11;13:21998. doi: 10.1038/s41598-023-49326-4 (PMC10713544; doi:10.1038/s41598-023-49326-4)
Supplement: Supplementary file 1 — Supplementary Information. [file 41598_2023_49326_MOESM1_ESM.docx]

**Appendix A**.- If non-maximally entangled photon sources are used in the new entanglement swapping protocol, the state that travels through the quantum channel is,

$|\zeta_{00}\rangle=\alpha|HH\rangle+\beta|VV\rangle$, (A1)

where $\left|\alpha\right|^{2}+\left|\beta\right|^{2}=1$, such that of a Hilbert’s space^6^. In the most general case $\alpha\neq\beta$. As in the case of Eq. (1), the relative phase ϕ is not considered in order not to complicate the following deduction. Therefore, the density matrix of the non-maximally entangled state of Eq. (A1) results,

$\rho_{|\zeta_{00}\rangle}=|\zeta_{00}\rangle\langle\zeta_{00}|=\left[\begin{matrix} \alpha\\ 0 \\ 0 \\ \beta\end{matrix}\right]\left[\begin{matrix} \alpha^{*} & 0 & 0 & \beta^{*} \end{matrix}\right]=\left[\begin{matrix} \left|\alpha\right|^{2} & 0 & 0 & \alpha\beta^{*} \\ 0 & 0 & 0 & 0 \\ 0 & 0 & 0 & 0 \\ \beta\alpha^{*} & 0 & 0 & \left|\beta\right|^{2} \end{matrix}\right]$. (A2)

Now, considering Fig. 3, and Eq. (5) from Eq. (5c), i.e., in the case of working with sources like those in Eq. (A1), we will have,

$|\psi\left(t_{2}\right)\rangle=|\zeta_{00}\rangle\otimes|\zeta_{00}\rangle,$ (A3)

$\begin{matrix} |\psi\left(t_{3}\right)\rangle=\left(I\otimes CNOT\otimes I\right)|\psi\left(t_{2}\right)\rangle\\ =\left(\left[\begin{matrix} 1 & 0 \\ 0 & 1 \end{matrix}\right]\otimes\left[\begin{matrix} 1 & 0 & 0 & 0 \\ 0 & 1 & 0 & 0 \\ 0 & 0 & 0 & 1 \\ 0 & 0 & 1 & 0 \end{matrix}\right]\otimes\left[\begin{matrix} 1 & 0 \\ 0 & 1 \end{matrix}\right]\right)|\zeta_{00}\zeta_{00}\rangle\\ =\left[\begin{matrix} \begin{matrix} \begin{matrix} \begin{matrix} \begin{matrix} \alpha^{2} & 0 & 0 & \alpha\beta\end{matrix} & 0 & 0 & 0 \end{matrix} & 0 & 0 & 0 \end{matrix} & 0 & 0 & 0 \end{matrix} & \beta^{2} & \alpha\beta& 0 \end{matrix}\right]^{T}, \end{matrix}$ (A4)

$\begin{matrix} |\psi\left(t_{4}\right)\rangle=\left(I\otimes H\otimes I\otimes I\right)|\psi\left(t_{3}\right)\rangle\\ =\left(\left[\begin{matrix} 1 & 0 \\ 0 & 1 \end{matrix}\right]\otimes\frac{1}{\sqrt{2}}\left[\begin{matrix} 1 & 1 \\ 1 & -1 \end{matrix}\right]\otimes\left[\begin{matrix} 1 & 0 & 0 & 0 \\ 0 & 1 & 0 & 0 \\ 0 & 0 & 1 & 0 \\ 0 & 0 & 0 & 1 \end{matrix}\right]\right)|\psi\left(t_{3}\right)\rangle\\ =\left[\begin{matrix} \begin{matrix} \begin{matrix} \begin{matrix} \begin{matrix} \alpha^{2} & 0 & 0 & \alpha\beta\end{matrix} & \alpha^{2} & 0 & 0 \end{matrix} & \alpha\beta& 0 & \beta^{2} \end{matrix} & \alpha\beta& 0 & 0 \end{matrix} & -\beta^{2} & -\alpha\beta& 0 \end{matrix}\right]^{T}/\sqrt{2}, \end{matrix}$ (A5)

and

$\begin{matrix} |\psi\left(t_{5}\right)\rangle=\left(I\otimes P_{h}\otimes P_{h}\otimes I\right)|\psi\left(t_{4}\right)\rangle\\ =\left(\left[\begin{matrix} 1 & 0 \\ 0 & 1 \end{matrix}\right]\otimes\left[\begin{matrix} 1 & 0 \\ 0 & 0 \end{matrix}\right]\otimes\left[\begin{matrix} 1 & 0 \\ 0 & 0 \end{matrix}\right]\otimes\left[\begin{matrix} 1 & 0 \\ 0 & 1 \end{matrix}\right]\right)|\psi\left(t_{4}\right)\rangle\\ =\left[\begin{matrix} \begin{matrix} \begin{matrix} \begin{matrix} \begin{matrix} \alpha^{2} & 0 & 0 & 0 \end{matrix} & 0 & 0 & 0 \end{matrix} & 0 & 0 & \beta^{2} \end{matrix} & 0 & 0 & 0 \end{matrix} & 0 & 0 & 0 \end{matrix}\right]^{T}/\sqrt{2}. \end{matrix}$ (A6)

Equation (A6) is totally concomitant with Eq. (5f), which highlights the robustness of the architecture proposed in this work when working with non-maximally entangled photon sources. In this way, the only change is that at the output of the configuration of Fig. 3, we will have a density matrix like that of Eq. (A2), instead of Eq. (4), which results from using maximally entangled photon sources.

The previous analysis is of main importance in real contexts where the entanglement swapping protocol must be used given that the entanglement suffers from the noise of the quantum channel, where the fidelity of entanglement is degraded. In this case, there are current novel solutions, which resort to entanglement purification^89,90^ to achieve a high-quality distillation of the entanglement in the presence of noise in the quantum channel.

The theoretical analysis of the impact of white noise as well as color noise^91^, both for the original version of the entanglement swapping protocol^69-75^ and for the proposed configuration, will be identical. We refer to the analysis based on the density operator of the Werner state^91^, which is represented as:

$\rho_{W}=p|\beta_{00}\rangle\langle\beta_{00}|+\Theta\left(p\right)\widehat{I}$, (A7)

where $\widehat{I}$ is a unit operator in four-dimensional space, *p* is a variable parameter and $\Theta$ is an operator that depends on the type of noise present in the quantum channel (white, color, or white and color), with $p\in\left[0,1\right]$, so that if *p* = 1, we have the pure Bell state, while if *p* = 0, we will have only noise.

Therefore, we can only resort to an experimental implementation of both protocols in the presence of noise to compare their performances. To carry out this experiment, we resorted to the configurations in Figs. 1 and 3, and the optical noise generator proposed in the paper of Jiang *et al*^92^, which will allow us to emulate optical noise in the short path in which the photons travel entangled by an optical fiber on the optical table. The change in the translation of the entangled photons via optical fiber is mandatory if we wish to use the optical noise generator of the paper of Jiang *et al*^92^, which essentially consists of a phase modulator controlled by a pseudo-random binary sequence generator with a bandwidth of 50 MHz. The cumulative dispersion of the fiber Bragg grating is set as *d* = 2000ps/nm, i.e., as in the experiment of Jiang *et al*^92^. Then, the results obtained were the following:

1. original protocol: Fidelity = 0.9114, and
2. proposed protocol: Fidelity = 0.9678.


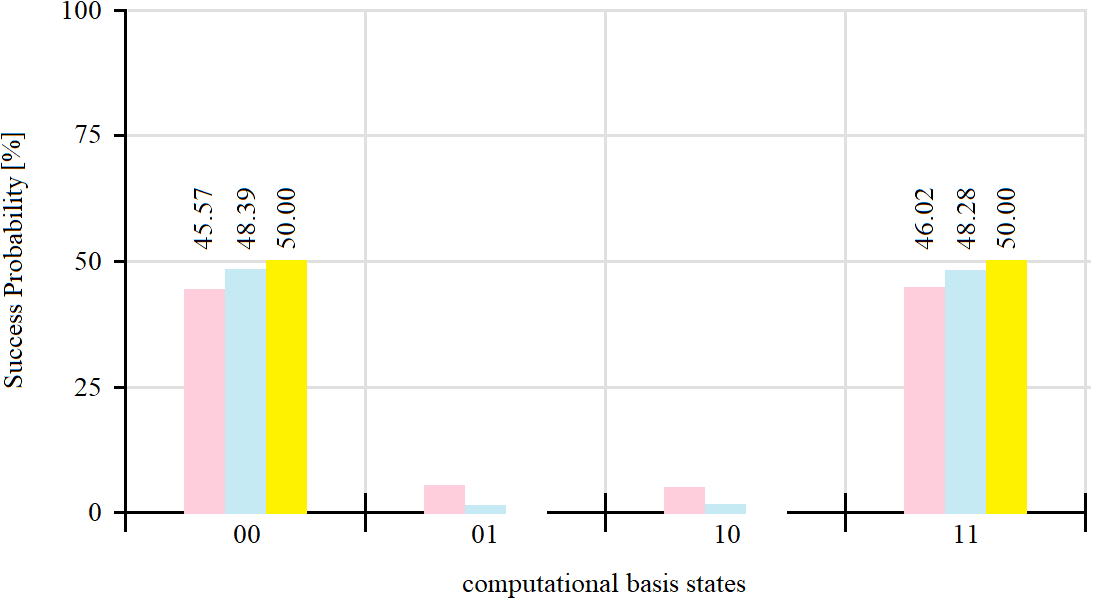


FIG. A1. Success probabilities of both protocols, where the pink bars correspond to the original protocol, the blue bars to the proposed, and the yellow bars to the case without noise.

The success probabilities of both protocols can be observed in Fig. A1, where the proposed protocol turns out to be more robust than the original protocol for the same perturbative conditions. Figure A1 shows the success probability of both protocols, in pink for the original one, and in blue for the proposed protocol, while the ideal noiseless case is represented in yellow.
